# Hawaiian Bobtail Squid Symbionts Inhibit Marine Bacteria via Production of Specialized Metabolites, Including New Bromoalterochromides BAC-D/D′

**DOI:** 10.1128/mSphere.00166-20

**Published:** 2020-07-01

**Authors:** Andrea M. Suria, Karen C. Tan, Allison H. Kerwin, Lucas Gitzel, Lydia Abini-Agbomson, Jessica M. Bertenshaw, Jaydeen Sewell, Spencer V. Nyholm, Marcy J. Balunas

**Affiliations:** a Department of Molecular and Cell Biology, University of Connecticut, Storrs, Connecticut, USA; b Division of Medicinal Chemistry, Department of Pharmaceutical Sciences, University of Connecticut, Storrs, Connecticut, USA; University of Wisconsin—Madison

**Keywords:** bacterial inhibition, *Euprymna scolopes*, accessory nidamental gland, jelly coat, *Pseudoalteromonas*, bromoalterochromides, defensive symbioses

## Abstract

The Hawaiian bobtail squid, Euprymna scolopes, has a symbiotic bacterial consortium in the accessory nidamental gland (ANG), a female reproductive organ that protects eggs against fouling microorganisms. To test the antibacterial activity of ANG community members, 19 bacterial isolates were screened for their ability to inhibit Gram-negative and Gram-positive bacteria, of which two strains were inhibitory. These two antibacterial isolates, *Leisingera* sp. ANG59 and *Pseudoalteromonas* sp. JC28, were subjected to further genomic characterization. Genomic analysis of *Leisingera* sp. ANG59 revealed a biosynthetic gene cluster encoding the antimicrobial compound indigoidine. The genome of *Pseudoalteromonas* sp. JC28 had a 14-gene cluster with >95% amino acid identity to a known bromoalterochromide (BAC) cluster. Chemical analysis confirmed production of known BACs, BAC-A/A′ (compounds 1a/1b), as well as two new derivatives, BAC-D/D′ (compounds 2a/2b). Extensive nuclear magnetic resonance (NMR) analyses allowed complete structural elucidation of compounds 2a/2b, and the absolute stereochemistry was unambiguously determined using an optimized Marfey’s method. The BACs were then investigated for *in vitro* antibacterial, antifungal, and nitric oxide (NO) inhibitory activity. Compounds 1a/1b were active against the marine bacteria Bacillus algicola and Vibrio fischeri, while compounds 2a/2b were active only against B. algicola. Compounds 1a/1b inhibited NO production via lipopolysaccharide (LPS)-induced inflammation in RAW264.7 macrophage cells and also inhibited the pathogenic fungus Fusarium keratoplasticum, which, coupled with their antibacterial activity, suggests that these polyketide-nonribosomal peptides may be used for squid egg defense against potential pathogens and/or fouling microorganisms. These results indicate that BACs may provide *Pseudoalteromonas* sp. JC28 an ecological niche, facilitating competition against nonsymbiotic microorganisms in the host’s environment.

**IMPORTANCE** Animals that deposit eggs must protect their embryos from fouling and disease by microorganisms to ensure successful development. Although beneficial bacteria are hypothesized to contribute to egg defense in many organisms, the mechanisms of this protection are only recently being elucidated. Our previous studies of the Hawaiian bobtail squid focused on fungal inhibition by beneficial bacterial symbionts of a female reproductive gland and eggs. Herein, using genomic and chemical analyses, we demonstrate that symbiotic bacteria from this gland can also inhibit other marine bacteria *in vitro*. One bacterial strain in particular, *Pseudoalteromonas* sp. JC28, had broad-spectrum abilities to inhibit potential fouling bacteria, in part via production of novel bromoalterochromide metabolites, confirmed via genomic annotation of the associated biosynthetic gene cluster. Our results suggest that these bacterial metabolites may contribute to antimicrobial activity in this association and that such defensive symbioses are underutilized sources for discovering novel antimicrobial compounds.

## Abstract (precis)

Animals that deposit eggs must protect their embryos from fouling and disease by microorganisms to ensure successful development. Although beneficial bacteria are hypothesized to contribute to egg defense in many organisms, the mechanisms of this protection are only recently being elucidated. Our previous studies of the Hawaiian bobtail squid focused on fungal inhibition by beneficial bacterial symbionts of a female reproductive gland and eggs. Herein, using genomic and chemical analyses, we demonstrate that symbiotic bacteria from this gland can also inhibit other marine bacteria *in vitro*. One bacterial strain in particular, *Pseudoalteromonas* sp. JC28, had broad-spectrum abilities to inhibit potential fouling bacteria, in part via production of novel bromoalterochromide metabolites, confirmed via genomic annotation of the associated biosynthetic gene cluster. Our results suggest that these bacterial metabolites may contribute to antimicrobial activity in this association and that such defensive symbioses are underutilized sources for discovering novel antimicrobial compounds.

## INTRODUCTION

Organisms that deposit eggs without subsequent parental care must have mechanisms to protect developing embryos from predation, infection, and fouling (1). To compensate, some insects utilize compounds produced by bacteria to defend against pathogens, e.g., beewolf wasps (2, 3) and houseflies (4). In marine environments, shrimp and lobsters supplement parental care with antifungal compounds produced by bacteria that coat their eggs (5, 6).

Many female cephalopods house a diverse bacterial consortium in a reproductive organ known as the accessory nidamental gland (ANG) (7–9). The ANG is adjacent to the nidamental glands, which produce a jelly that surrounds developing embryos that are laid externally in egg clutches. Bacteria from the ANG are deposited into this jelly coat (JC), where they are hypothesized to produce bioactive metabolites that defend the eggs from bacterial and fungal infections or competitively exclude pathogens from settling on the egg surface (10, 11). In the Hawaiian bobtail squid, Euprymna scolopes, these ANG bacteria are deposited into the JC surrounding developing embryos to protect eggs from fungal fouling during an approximately month-long embryonic period (11).

In E. scolopes, the ANG and JC communities are composed of *Alphaproteobacteria*, *Verrucomicrobia*, *Gammaproteobacteria*, and *Flavobacteriia* (9, 12). Genomic analyses of some *Alphaproteobacteria* isolates revealed the potential to produce secondary metabolites, including utilization of polyketide synthase (PKS) or nonribosomal peptide synthetase (NRPS) pathways, among others (13, 14). A JC isolate, *Leisingera* sp. JC1, inhibited several vibrio bacteria *in vitro*, in part due to production of the antimicrobial pigment indigoidine (14). In a subsequent study, the ANG isolate *Labrenzia* sp. ANG18 was found to produce lincomycin B, a derivative of the antibacterial drug lincomycin A, and both metabolites were detected in an egg clutch extract (11). Butanol extracts from *Gammaproteobacteria* ANG and JC isolates of a different squid, Doryteuthis pealeii (formerly Loligo pealeii), inhibited *Vibrio*, *Aeromonas*, *Staphylococcus*, *Streptomyces*, and Escherichia coli strains in a disc diffusion assay (15). Given that symbiont-derived antimicrobial metabolites are implicated in the cephalopod ANG symbiosis, we sought to further investigate the antibacterial response of ANG and JC bacteria from *E. scolopes*.

In this study, we screened 19 ANG and JC bacterial isolates representing *Alphaproteobacteria*, *Gammaproteobacteria*, and *Flavobacteriia* for their ability to inhibit Gram-positive and Gram-negative marine bacteria. Genomic analyses were utilized to analyze the biosynthetic potential of two bioactive isolates, *Leisingera* sp. ANG59 and *Pseudoalteromonas* sp. JC28. We describe a bromoalterochromide gene cluster from *Pseudoalteromonas* sp. JC28 and the isolation and complete structural characterization of new bromoalterochromide (BAC) derivatives. Additional bioactivity profiling was conducted on purified BACs to understand how these bacterial secondary metabolites might contribute to the ANG symbiosis.

## RESULTS

### Zone of inhibition assays.

An initial screen of the *in vitro* antibacterial activity of 19 ANG and JC isolates revealed that two strains, *Leisingera* sp. ANG59 and *Pseudoalteromonas* sp. JC28, inhibited one or more of the marine bacteria tested (Table 1; see also Table S3a in the supplemental material). All other ANG/JC strains tested did not form zones of inhibition (ZOIs) when spotted on the surfaces of marine Gram-negative and Gram-positive lawns at a high density (10^8^ CFU/ml). The *Alphaproteobacteria* strain, *Leisingera* sp. ANG59, produced moderate zones of inhibition against two of the vibrio strains, Vibrio fischeri and Vibrio anguillarum. These results are comparable to those observed for an E. scolopes jelly coat isolate, *Leisingera* sp. JC1, which inhibited select vibrio strains in a similar bioassay (14). *Leisingera* sp. ANG59 produced a dark-blue pigment, and our genome analyses revealed an indigoidine biosynthetic gene cluster (Table S4), likely associated with antimicrobial activity similar to what was described for *Leisingera* sp. JC1 (14).

**TABLE 1:** *In vitro* antibacterial activity of ANG/JC isolates against seven marine bacteria

Taxon and ANG/JC isolate	Zone of inhibition for the following strain^a^:						
	1	2	3	4	5	6	7
*Alphaproteobacteria*							
*Leisingera* sp. ANG1	−	−	−	−	−	−	−
*Leisingera* sp. ANG-M6	−	−	−	−	−	−	−
*Leisingera* sp. ANG-DT	−	−	−	−	−	−	−
*Leisingera* sp. ANG-S	−	−	−	−	−	−	−
*Leisingera* sp. ANG-S3	−	−	−	−	−	−	−
*Leisingera* sp. ANG52	−	−	−	−	−	−	−
*Leisingera* sp. ANG59	+	+	−	−	−	−	−
*Leisingera* sp. JC11	−	−	−	−	−	−	−
*Rhodobacteraceae* sp. ANG7	−	−	−	−	−	−	−
*Rhodobacteraceae* sp. ANG13	−	−	−	−	−	−	−
*Labrenzia* sp. ANG18	−	−	−	−	−	−	−
*Ruegeria* sp. ANG6	−	−	−	−	−	−	−
*Ruegeria* sp. ANG10	−	−	−	−	−	−	−
*Ruegeria* sp. ANG-S4	−	−	−	−	−	−	−

*Flavobacteriia*							
*Muricauda* sp. ANG21	−	−	−	−	−	−	−

*Gammaproteobacteria*							
*Shewanella* sp. ANG44	−	−	−	−	−	−	−
*Alteromonas* sp. JC21	−	−	−	−	−	−	−
*Pseudoalteromonas* sp. JC28	+	+	+	+	+	+	+
*Vibrio* sp. JC34	−	−	−	−	−	−	−

a Symbols: +, zone of inhibition observed; −, no zone of inhibition observed (results from three trials). The numbers refer to the following strains: 1, Vibrio fischeri ES114; 2, Vibrio anguillarum 775; 3, Vibrio parahaemolyticus KNH1; 4, Vibrio harveyi B392; 5, Photobacterium leiognathi KNH6; 6, Bacillus megaterium CNJ778; 7, Exiguobacterium aestuarii CNJ771.

**FIG 1:**
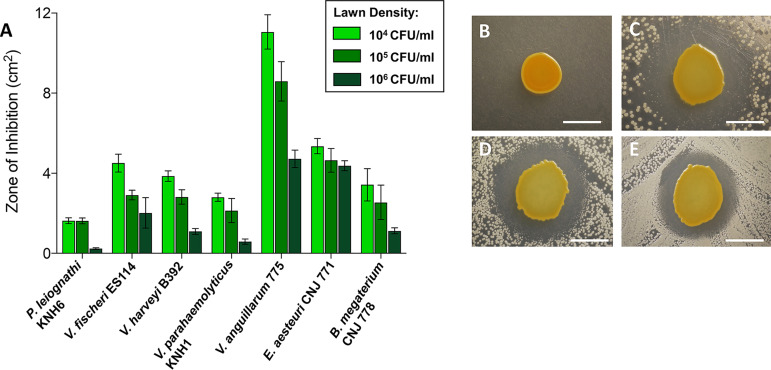
*Pseudoalteromonas* sp. JC28 inhibits Gram-positive and Gram-negative marine bacteria. (A) Zones of inhibition (areas in square centimeters) were measured on lawns of marine bacteria with increasing lawn densities. Significantly greater inhibition was noted between 10^4^ and 10^5^ CFU/ml and between 10^5^ and 10^6^ CFU/ml densities of all species with the exception of the 10^4^ to 10^5^ CFU/ml densities of *P. leiognathi* and the 10^5^–10^6^ CFU/ml densities of *E. aesteuri* (*post hoc* Tukey tests from two-way ANOVA). Data are presented as means ± standard deviations (error bars) for nine trials. (B to E) Representative images of JC28 monoculture control (B) and JC28 cocultured with *P. leiognathi* at 10^4^ CFU/ml (C), 10^5^ CFU/ml (D), and 10^6^ CFU/ml (E) lawn densities. Bars = 5 mm.

The *Gammaproteobacteria* JC isolate, *Pseudoalteromonas* sp. JC28, showed the strongest activity of all isolates tested and was able to inhibit all seven target bacteria, including five Gram-negative strains and two Gram-positive strains. We previously showed that *Pseudoalteromonas* sp. JC28 inhibited two types of fungi, Fusarium keratoplasticum and Candida albicans (11). Due to this broad-spectrum inhibitory activity, JC28 was selected for further antibacterial testing to determine whether greater inhibition was observed at lower target lawn densities. Testing lower lawn densities (10^4^ to 10^6^ CFU/ml) would be more representative of the concentrations of bacteria in natural seawater conditions where these isolates are exposed during the course of egg development. Significantly greater inhibition was observed at lawn densities between 10^4^ and 10^5^ CFU/ml and between 10^5^ and 10^6^ CFU/ml for all test strains except for Photobacterium leiognathi and Exiguobacterium aesteuri (Fig. 1). The greatest inhibition was against V. anguillarum (ZOI = 11.1 cm^2^; Table S3b) followed by E. aesteuri (ZOI = 5.3 cm^2^; Table S3b).

### Genomic and phylogenetic analyses. (i) *Leisingera* sp. ANG59.

The draft genome of *Leisingera* sp. ANG59 was assembled into 109 scaffolds with a genome size of 5.33 Mb, GC content of 62.9%, *N*_50_ of 121,837 bp, and 5,169 coding sequences. The genome contained all genes to utilize glucose through the Entner-Doudoroff pathway and tricarboxylic acid (TCA) cycle. Due to the absence of a phosphofructokinase ortholog, the Entner-Duodoroff or pentose-phosphate pathways are likely utilized instead of glycolysis. Nitrate and nitrite reductases formed a complete denitrification pathway. The essential structural genes were present for the type II, type IV, and type VI secretion systems. Flagellar biosynthesis and chemotaxis genes were present, suggesting that ANG59 may be capable of motility in response to chemoattractants. The antiSMASH analysis predicted seven secondary metabolite biosynthetic gene clusters, including two siderophore clusters, two “other” clusters, one bacteriocin cluster, one homoserine lactone cluster, and one ectoine cluster (Table S5). The “other” cluster present on scaffold 69.1 contained the six genes necessary for biosynthesis of the antimicrobial pigment indigoidine with ≥75% amino acid similarity (Table S4) to the indigoidine genes present in the previously described *E. scolopes* JC isolate, *Leisingera* sp. JC1 (14). The homoserine lactone cluster contained the autoinducer synthase gene, *raiI*, and the transcriptional activator, *raiR*, similar to the clusters present in several other ANG isolates (13), although it is still unknown which genes are regulated by this quorum sensing system.

### (ii) *Pseudoalteromonas* sp. JC28.

**FIG 2:**
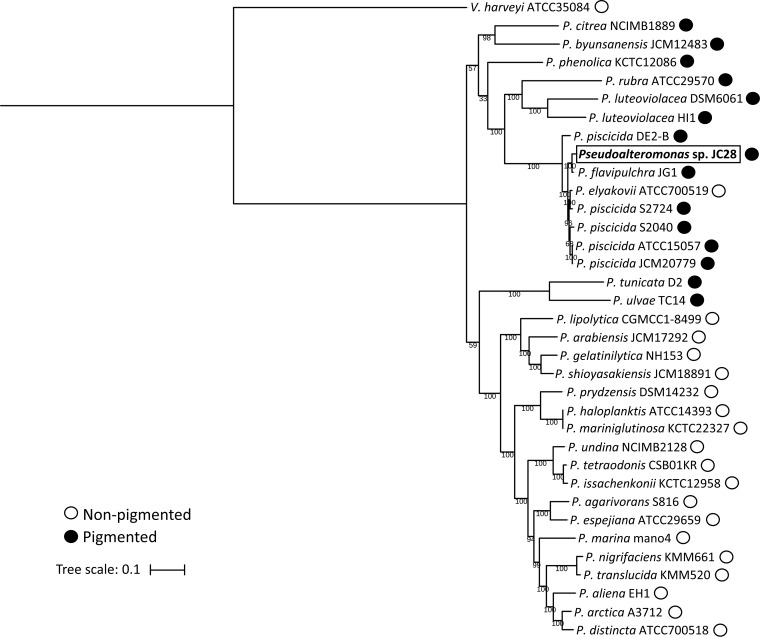
MLSA analysis of *Pseudoalteromonas* genomes and the *Pseudoalteromonas* sp. JC28 isolate. Phylogenetic analysis was performed with six single-copy housekeeping genes. Isolate *Pseudoalteromonas* sp. JC28 (bold and boxed) falls within a clade containing the pigmented *P. flavipulchra* JG1. Numbers on branches indicate bootstrap values. Black circles indicate pigmented strains, and white circles indicate nonpigmented strains.

The draft genome of *Pseudoalteromonas* sp. JC28 was assembled into 40 scaffolds with a genome size of 5.53 Mb, GC content of 43.2%, *N*_50_ of 509,001 bp, and 4,894 coding sequences. In the multilocus sequence analysis (MLSA), *Pseudoalteromonas* sp. JC28 was placed in a well-supported clade containing mostly pigmented pseudoalteromonads isolated from seawater samples (Fig. 2). The closest strain to JC28 was Pseudoalteromonas flavipulchra JG1. This clade also contained five Pseudoalteromonas piscicida strains and one Pseudoalteromonas elyakovii strain. With a few exceptions, most strains examined fell into either a pigmented or nonpigmented clade, as has been previously described (16).

Genome analyses of *Pseudoalteromonas* sp. JC28 revealed all of the genes necessary to utilize glucose through glycolysis and the Entner-Doudoroff pathway, pentose phosphate pathway, and the TCA cycle. Genes for the assimilatory sulfate reduction pathway were detected, as well as ammonia assimilation into glutamate. For secretion systems, genes for the type IV and type VI secretion systems were present. Flagellar biosynthesis, motility, and many of the key chemotaxis genes were also found. While only one of the four genes to make the siderophore aerobactin (*iucB*) was detected, the TonB-dependent receptor (*iutA*) and siderophore transport genes (*fhuC* and *exB*) were present, suggesting that JC28 may be able to utilize iron-chelating siderophores produced by neighboring bacteria under iron-limited conditions (17, 18).

Analysis of the JC28 genome with antiSMASH revealed a total of 18 predicted secondary metabolite gene clusters. These gene clusters included nine nonribosomal peptide synthetase (NRPS) clusters, four hybrid type 1 polyketide synthase-NRPS (T1PKS-NRPS) clusters, one hybrid ladderane-NRPS cluster, three bacteriocin clusters, and one thiopeptide cluster (Table S5). Two of the predicted NRPS clusters in the JC28 genome were found at the end of a contig, and five NRPS clusters encompassed an entire contig (Table S5), suggesting that the number of clusters may be inflated due to breaks in the assembly at these regions.

**FIG 3:**
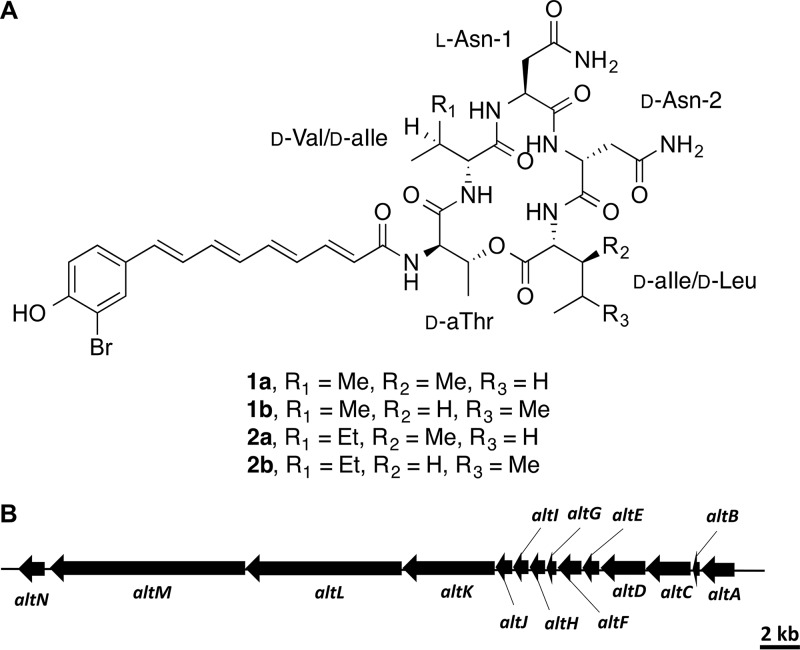
Complete characterization of bromoalterochromides BAC-A/A′ (compounds 1a/1b) and BAC-D/D′ (compounds 2a/2b). (A) Structures of bromoalterochromides BAC-A/A′ (compounds 1a/1b) and BAC-D/D′ (compounds 2a/2b). (B) Bromoalterochromide biosynthetic operon.

The predicted ladderane-NRPS cluster had 100% amino acid similarity to a known bromoalterochromide (BAC)-producing cluster from P. piscicida JCM20779 (19). This operon contains 14 genes, including three NRPS genes (*altKLM*) and a halogenase gene (*altN*) which attaches the bromine to the alterochromide (Fig. 3B and Table 2), as has been previously proposed for BAC biosynthesis (20). Bromoalterochromides are yellow lipopeptides known to have cytotoxic activity on sea urchin eggs (21) and the ciliate Tetrahymena pyriformis (22), as well as antibacterial activity against Bacillus subtilis (19). Within the pigmented group, all strains in the branch that contained JC28 also contained a BAC biosynthetic gene cluster (Table S6). Of the other known secondary metabolite biosynthetic pathways reported for pseudoalteromonads, *Pseudoalteromonas* sp. JC28 also has the antibacterial l-amino acid oxidase gene, *pfaP* (96% amino acid similarity, 100% query cover), produced by P. flavipulchra JG1 (23). We did not detect genes for production of bromopyrroles/bromophenols (24), tambjamine YP1 (25), thiomarinol (26), or violacein (27, 28), which have been previously reported in other *Pseudoalteromonas* species.

**TABLE 2:** Comparison of the bromoalterochromide biosynthetic gene cluster in *Pseudoalteromonas* sp. JC28 and *P. piscicida* JCM20779^*a*^

Gene	Gene function	*Pseudoalteromonas* sp. JC28 locus ID	*P. piscicida* locus ID	% amino acid similarity to *P. piscicida* operon	E value	% query cover
*altN*	Flavin-dependent halogenase	DS891_00345	PpisJ2_10100002431	97.82	0	100
*altM*	Nonribosomal peptide synthetase	DS891_00350	PpisJ2_10100002426	96.89	0	100
*altL*	Nonribosomal peptide synthetase	DS891_00355	PpisJ2_10100002421	97.28	0	100
*altK*	Nonribosomal peptide synthetase	DS891_00360	PpisJ2_10100002416	98.42	0	100
*altJ*	Thioesterase	DS891_00365	PpisJ2_10100002411	98.01	0	100
*altI*	Hypothetical protein	DS891_00370	PpisJ2_10100002406	95.74	9e−171	100
*altH*	SDR family NAD(P)-dependent oxidoreductase	DS891_00375	PpisJ2_10100002401	99.18	0	100
*altG*	3-Hydroxydecanoyl- ACP dehydratase	DS891_00380	PpisJ2_10100002396	100	6e−99	100
*altF*	ABC transporter permease	DS891_00385	PpisJ2_10100002391	100	0	100
*altE*	ABC transporter ATP-binding protein	DS891_00390	PpisJ2_10100002386	100	0	100
*altD*	3-Oxoacyl-ACP synthase	DS891_00395	PpisJ2_10100002381	97.66	0	100
*altC*	Acyl-CoA synthetase/ AMP-fatty acid ligase	DS891_00400	PpisJ2_10100002376	97.99	0	100
*altB*	Acyl carrier protein	DS891_00405	PpisJ2_10100002371	100	3e−68	100
*altA*	Aromatic amino acid lyase	DS891_00410	PpisJ2_10100002366	99.63	0	100

a Abbreviations: ID, identifier; ACP, acyl carrier protein; acyl-CoA, acyl coenzyme A.

### Isolation and structure elucidation of bromoalterochromides (BACs).

**FIG 4:**
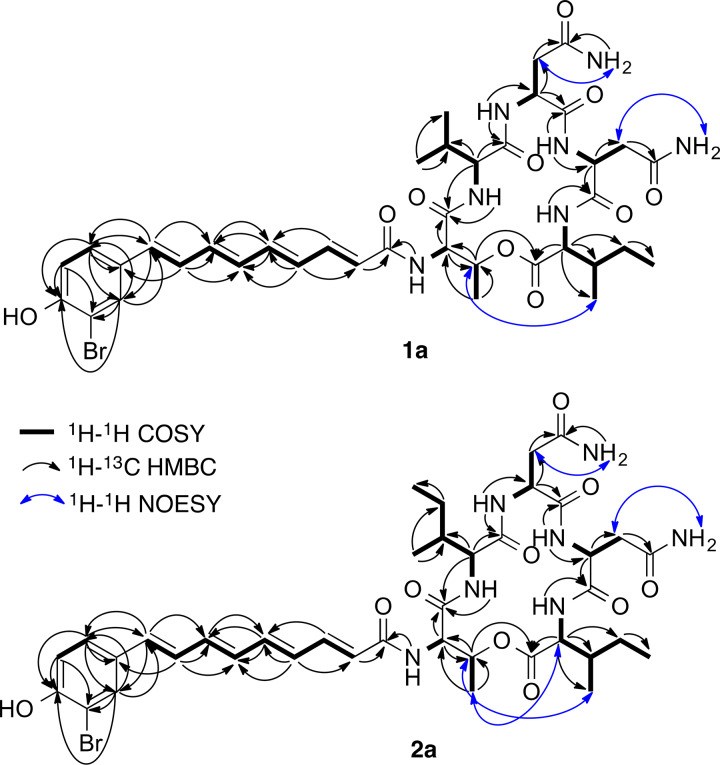
Two-dimensional NMR correlations of bromoalterochromides BAC-A (compound 1a) and BAC-D (compound 2a). Data obtained from ^1^H-^1^H COSY, ^1^H-^13^C HMBC, and ^1^H-^1^H NOESY spectra (Fig. S2 and S3).

Organic extracts obtained from both the supernatant and pellet of JC28 cultures were analyzed by liquid chromatography-mass spectrometry (LC-MS) (Table S7), with the ethyl acetate (EtOAc) supernatant extract and the methanol (MeOH) pellet extract exhibiting several peaks with strong absorption at 395 nm, with MS spectra characteristic of brominated compounds (data not shown). These two extracts were then purified separately by reverse-phase high-performance liquid chromatography (RP-HPLC), resulting in a combined total of 4.1 mg of compounds 1a/1b and 1.1 mg of compounds 2a/2b. High-resolution (HR) MS analysis of compounds 1a/1b and compounds 2a/2b showed [M+H]^+^ peaks *m/z* 844.2899 and *m/z* 858.3034, respectively, which both agree with the calculated [M+H]^+^, 844.2881 and 858.3037, for C_38_H_51_BrN_7_O_10_ and C_39_H_53_BrN_7_O_10_. This also indicated that compounds 2a/2b have an additional CH_2_ unit compared to compounds 1a/1b. Extensive analysis by NMR spectroscopy revealed that compounds 1a/1b are an inseparable mixture of constitutional isomers of the known BACs (Fig. 3A; see also Fig. S2 in the supplemental material), BAC-A/A′, having spectroscopic data superimposable with those in the literature (21). Furthermore, compounds 2a/2b were determined to be another pair of inseparable isomers of two new BAC derivatives, which we have named BAC-D/D′ (Fig. 3A and Fig. S3). Although two studies have previously detected these new derivatives from *P. piscicida* JCM20779 and P. maricaloris KMM636 (20, 29), their actual structures have never been reported. Much of the heteronuclear multiple-bond correlation (HMBC) and nuclear Overhauser effect spectroscopy (NOESY) NMR data of compound 2a were identical with those of compound 1a, indicating that the connectivity between the five amino acid residues and the acyl chain are the same (Fig. 4, Fig. S2d and e, and Fig. S3d and e). The ^1^H NMR spectra of the two pairs of derivatives were nearly identical, except that the proton signals at 3.96 and 1.87 ppm (Table 3), assigned to H-2 and H-3 of the Val moiety in compounds 1a/1b, could not be observed in the ^1^H NMR spectrum of compounds 2a/2b (Fig. S2a and Fig. 3a). This suggested that the Val residue should be replaced with either an Ile or Leu in compounds 2a/2b. Correlation spectroscopy (COSY) correlations (Fig. S3b and Table 3) between a CH proton at 1.68 ppm and doublet CH_3_ protons at 0.90 ppm and between CH_2_ protons at 1.13 and 1.42 ppm confirmed that compounds 2a/2b possess an Ile moiety in place of Val, resulting in determination of the planar structure of compounds 2a/2b (Fig. 3A).

**TABLE 3:** ^1^H and ^13^C chemical shifts of compounds 1a/1b and 2a/2b in DMSO-*d_6_* (600 and 125 MHz, respectively)^*a*^

Component	Compound 1a		Compound 1b		Compound 2a		Compound 2b	
	δ_H_, multiplicity (^3^J_H-H_)	δ_C_	δ_H_, multiplicity (^3^J_H-H_)	δ_C_	δ_H_, multiplicity (^3^J_H-H_)	δ_C_	δ_H_, multiplicity (^3^J_H-H_)	δ_C_
d-Val/*allo*-Ile-1								
NH	8.18, d (6.0)		8.18, d (6.0)		8.11, m		8.11, m	
1		171.9		171.9		172.2		172.2
2	3.96, m	59.0	3.96, m	59.0	4.18, m	57.1	4.18, m	57.1
3	1.87, m	29.5	1.87, m	29.5	1.68, m	35.8	1.68, m	35.8
3-Me	0.88, d (6.0)	18.6	0.88, d (6.0)	18.6	0.90, d (6.0)	14.8	0.90, d (6.0)	14.8
4	0.95, d (6.0)	18.9	0.95, d (6.0)	18.9	1.13, m	25.6	1.13, m	25.6
					1.43, m		1.43, m	
5					0.84, m	11.3	0.84, m	11.3

l-Asn-1								
NH	8.65, d (6.0)		8.70, d (6.0)		8.64, d (6.0)		8.67, d (6.0)	
1		171.4		170.2		171.4		170.3
2	4.32, m	51.1	4.30, m	51.1	4.30, m	51.7	4.28, m	51.7
3	2.41, m	35.8	2.41, m	35.8	2.41, m	36.1	2.41, m	36.1
4		170.7		170.7		170.7		170.7
NH_2_	6.90, br		6.90, br		6.91, br		6.91, br	
	7.28, br		7.28, br		7.30, br		7.30, br	

d-Asn-2								
NH	8.11, br		7.70, d (6.0)		8.12, m		7.73, d (6.0)	
1		170.0		170.2		170.0		170.3
2	4.39, m	50.6	4.43, m	50.6	4.39, m	50.7	4.43, m	50.7
3	2.74, dd (18.0,6.0)	35.1	2.55, m	35.1	2.75, dd (18.0,6.0)	35.2	2.55, m	36.0
4		172.2		170.2		172.2		172.2
NH_2_	6.82, br		6.82, br		6.82, br		6.82, br	
	7.25, br		7.25, br		7.30, br		7.30, br	

d-*allo*-Ile/Leu								
NH	7.15, m		8.36, d (6.0)		7.22, m		8.35, d (6.0)	
1		169.3		171.1		169.3		171.1
2	4.36, m	55.8	4.43, m	49.7	4.35, m	56.4	4.45, m	49.7
3	1.96, m	36.4	1.65, m	38.7	1.99, m	36.6	1.64, m	38.4
			1.88, m				1.88, m	
3-Me/4-Me	0.80, d (6.0)	14.3	0.84, m	21.4	0.80, d (6.0)	14.6	0.84, m	21.9
4	1.05, m	25.7	1.63, m	23.7	1.05, m	25.7	1.63, m	23.7
	1.33, m				1.33, m			
5	0.89, t (6.0)	11.1	0.91, d (6.0)	23.7	0.89, t (6.0)	11.3	0.91, d (6.0)	23.7

d-*allo*-Thr								
NH	8.25, d (6.0)		8.25, d (6.0)		8.28, m		8.28, m	
1		168.8		169.2		168.8		168.8
2	4.74, m	54.8	4.82, m	54.8	4.77, m	55.0	4.77, m	55.0
3	4.75, m	72.2	4.75, m	72.2	4.76, m	72.3	4.76, m	72.3
3-Me	1.36, d (6.0)	16.6	1.36, d (6.0)	16.6	1.34, d (6.0)	16.5	1.34, d (6.0)	16.5

Acyl								
1		164.7		164.7		164.8		164.8
2	6.19, d (12.0)	123.7	6.19, d (12.0)	123.7	6.20, d (12.0)	123.8	6.20, d (12.0)	123.8
3	7.14, m	139.6	7.14, m	139.6	7.13, m	139.6	7.13, m	139.6
4	6.42, m	129.9	6.42, m	129.9	6.40, m	129.9	6.40, m	129.9
5	6.72, dd (18.0, 12.0)	139.1	6.72, dd (18.0, 12.0)	139.1	6.72, dd (18.0, 12.0)	139.3	6.72, dd (18.0, 12.0)	139.3
6	6.47, m	131.7	6.47, m	131.7	6.45, m	131.5	6.45, m	131.5
7	6.56, dd (18.0, 12.0)	136.2	6.56, dd (18.0, 12.0)	136.2	6.54, m	136.4	6.54, m	136.4
8	6.86, m	126.9	6.86, m	126.9	6.89, m	126.9	6.89, m	126.9
9	6.58, d (18.0)	132.6	6.58, d (18.0)	132.6	6.58, m	132.4	6.58, m	132.4
1′		129.8		129.8		129.7		129.7
2′	7.65, s	130.6	7.65, s	130.6	7.64, s	130.6	7.64, s	130.6
3′		109.8		109.8		109.8		109.8
4′		153.9		153.9		154.2		154.2
4′-OH	nd		nd		nd		nd	
5′	6.90, m	116.3	6.90, m	116.3	6.92, m	116.3	6.90, m	116.3
6′	7.32, m	126.7	7.32, m	126.7	7.32, m	126.7	7.32, m	126.7

a nd, not detected.

During the course of structure determination, we noted several chemical shift assignments on the aromatic ring carbons (C-1′, C-2′, and C-6′) of BAC-A/A′ compounds 1a/1b that were incongruent with the previous assignments (21), necessitating their reassignment herein (Table 3 and Fig. S2). Moreover, because of the higher spectroscopic resolution used in the present study, the chemical shifts for all olefins in compounds 1a/1b were definitively assigned.

We set out to confirm the absolute configuration of BAC-A/A′ compounds 1a/1b and determine that for BAC-D/D′ compounds 2a/2b. Several inconsistencies in the absolute configuration of BAC-A/A′ can be found in the literature. By chiral gas chromatography (GC)-MS analysis of the derivatized acid hydrolysate, all five amino acids in the peptide portion of BAC-A/A′ were tentatively assigned as having l configurations and the Thr residue was given a *threo* assignment (21). These stereochemical assignments are in opposition to those previously reported (30) and predicted by the bioinformatic data obtained from the *P. piscicida* JCM20779 and *Pseudoalteromonas* sp. JC28 BAC gene clusters, wherein all NRPS modules bear an epimerase domain, except for that of Asn-1, and the module for Thr was predicted to favor the *erythro* diastereomer (19, 20).

To determine the absolute stereochemistry, the C_3_ Marfey’s method was performed on the acid hydrolysates of compounds 1a/1b and 2a/2b, as well as on all isomers of the standard amino acids (31). In addition, since Asn was expected to be converted, in part or completely, to Asp under the hydrolytic conditions applied to the natural products (32), standards of Asp were derivatized and analyzed in the same manner. Analyzing the 1-fluoro-2,4-dinitrophenyl-5-l-leucinamide (l-FDLA)-derived samples by LC-MS, compounds 1a/1b were found to bear d-Val, l- and d-Leu (1:6), d-Ile or d-*allo*-Ile, d-*allo*-Thr, and l- and d-Asp (1:2) (Table 4). No peak corresponded to Asn, indicating complete hydrolysis to Asp. Since both isomers of Asp were detected from the hydrolysate, Asn-1 and Asn-2 were assigned to be in the l form and d form, respectively, in accordance with the JC28 genomic data. However, under the employed analytical conditions, d-Ile and d-*allo*-Ile could not be fully resolved. Consequently, 1-fluoro-2,4-dinitrophenyl-5-l-alaninamide (l-FDAA)-derived samples were prepared and analyzed using LC-MS, where d-*allo*-Ile, rather than d-Ile, was observed in the compound 1a/1b hydrolysate. Similar results were acquired for compounds 2a/2b, with the exception that a Val peak was not detected. Therefore, the complete structures were designated d-*allo*-Thr−d-Val−l-Asn1−d-Asn2−d-*allo*-Ile/d-Leu for compounds 1a/1b, and d-*allo*-Thr−d-*allo*-Ile−l-Asn1−d-Asn2−d-*allo*-Ile/d-Leu for compounds 2a/2b.

**TABLE 4:** Retention times (*t_R_*) of standards and hydrolysate derivatives using l-FDLA^*a*^ or l-FDAA^*b*^

Amino acid	*t_R_* (min)		
	Standards	Compounds 1a/1b	Compounds 2a/2b
l-Val	30.01	nd	nd
d-Val	37.09	37.12	nd
l-Leu	31.55	31.57	nd
d-Leu	40.10	40.13	40.58
l-Ile	31.37	nd	nd
d-Ile	39.27	39.27	39.66
l-*allo*-Ile	30.81	nd	nd
d-*allo*-Ile	39.21	39.27	39.66
l-Thr	21.70	nd	nd
d-Thr	28.52	nd	nd
l-*allo*-Thr	23.42	nd	nd
d-*allo*-Thr	25.95	25.96	26.26
l-Asp	23.97	23.97	24.22
d-Asp	27.16	27.13	27.54
d-Ile^*b*^	73.64	nd	nd
d-*allo*-Ile^*b*^	74.33	74.67	74.80

a FDLA-derived samples were analyzed by a linear gradient from 30% mobile phase B to 100% mobile phase B over 55 min, using 5% CH_3_CN with 1% HCOOH in water as mobile phase A and 5% CH_3_CN with 1% HCOOH in MeOH as mobile phase B. nd, not detected.

b FDAA-derived samples were analyzed by a linear gradient from 12.5% mobile phase B to 57.5% mobile phase B over 80 min, using 7.5% CH_3_CN with 1% HCOOH in water as mobile phase A and 7.5% CH_3_CN with 1% HCOOH in MeOH as mobile phase B. nd, not detected.

### Functional biological assays.

To investigate the potential functional role of these PKS-NRPS metabolites, BAC-A/A′ 1a/1b and BAC-D/D′ 2a/2b were tested against two marine bacteria, the Gram-positive strain Bacillus algicola CNJ 803 and the Gram-negative strain V. fischeri ES114. BAC-A/A′ (1a/1b) exhibited an MIC of 7.4 μM against B. algicola and 59.3 μM against V. fischeri. BAC-D/D′ (2a/2b) had a similar MIC of 7.3 μM against *B. algicola* but without activity against V. fischeri (MIC of >58.3 μM). Interestingly, both BAC isolates were more active against *B. algicola* than V. fischeri.

Since previous work showed that *Pseudoalteromonas* sp. JC28 strongly reduced fungal hyphal growth of Fusarium keratoplasticum FSSC-2g (11), BAC-A/A′ 1a/1b were tested to determine whether BACs may be, at least, partly responsible for the observed activity (2a/2b were not tested due to scarcity of material). BAC-A/A′ 1a/1b exhibited a 28% reduction in the surface area of F. keratoplasticum hyphal growth, in comparison with the negative control. BAC-A/A′ 1a/1b were then subjected to a nitric oxide (NO) inhibition assay to understand potential interactions with the host immune response. Coincubation of lipopolysaccharide (LPS)-treated RAW264.7 macrophage cells with 1a/1b resulted in inhibition of NO production with a 50% inhibitory concentration (IC_50_) of 16.9 μM.

## DISCUSSION

The ANG association of cephalopods has been hypothesized to act as a defensive symbiosis for egg protection, and our previous work showed that many ANG and JC bacterial isolates from *E. scolopes* inhibit fungi (11) and another JC isolate inhibited a number of marine vibrios (14). Although many of the isolates in this study failed to inhibit other marine bacteria *in vitro*, *Leisingera* sp. ANG59 inhibited some strains, and *Pseudoaltermonas* sp. JC28 showed broad-spectrum antibacterial activity. Our initial screen using a high target lawn concentration aimed to reveal ANG and JC isolates with strong antimicrobial activity. Future work will include screening at lower target lawn densities and other conditions that may reveal the inhibitory potential of other strains in different growth environments. Bacteria in the eggs may also have synergistic antibacterial activity as has been observed of *in vitro* antifungal activity in the bacterial symbionts of *Hydra* (33), and thus, future screens should also test bacterial cocultures. Interestingly, the most inhibitory strain, *Pseudoalteromonas* sp. JC28, is from a genus of relatively low abundance in the ANG, while *Leisingera* sp. ANG59 is from one of the most abundant genera detected and showed only moderate inhibition (12). Recent reviews have highlighted the importance rare members can have on microbial community composition and function (34, 35). For example, removal of low-abundance bacteria in soil communities can lead to the loss of antifungal volatiles that suppress the root pathogen Fusarium oxysporum (36).

The *Pseudoalteromonas* genus comprises Gram-negative, heterotrophic, and aerobic marine bacteria that belong to the subclass of *Gammaproteobacteria* (16, 37, 38). The pseudoalteromonads demonstrate a broad range of antimicrobial, antisettlement, and cytotoxic activity that is thought to be attributed mainly to the pigmented members of this group (16). *Pseudoalteromonas* sp. JC28 belongs to the pigmented group, and here we show that it has antibacterial activity against a number of marine bacteria in addition to previously reported antifungal activity (11). Within the branch containing *Pseudoalteromonas* sp. JC28, many *P. piscicida* strains have also shown strong inhibition against vibrios and other bacterial pathogens (39, 40) and antifungal activity against several fungi, including *Arthrinium* c.f. *saccharicola* (41) and Fusarium oxysporum (42). *P. piscicida* strains have also demonstrated eukaryotic toxicity, killing Artemia nauplii brine shrimp and Caenorhabditis elegans nematodes (43), and lysing Gymnodinium catenatum algal cells (44). Other *P. piscicida* strains inhibited settlement of red algae (*Polysiphonia* sp.) and green algae (Ulva lactuca) spores (39, 45).

*Pseudoalteromonas* species play important roles in interacting with marine invertebrates and are necessary for settlement or induction of morphogenesis in some invertebrate larvae, such as the polychaete Hydroides elegans (46), the coral Acropora millepora (47), or the sea urchin Heliocidaris erythrogramma (48). *Pseudoalteromonas* sp. JC28 does not have the settlement genes associated with Pseudoalteromonas luteoviolacea HI1 (49), but future research may test whether it produces compounds that influence invertebrate larval or algal settlement, for example, in preventing fouling of eggs.

The closest relative of *Pseudoalteromonas* sp. JC28 strain, *P. flavipulchra* JG1, was isolated from the rearing water of healthy turbot in Qingdao, China, and was investigated as a potential probiotic for fish aquaculture (50). It strongly inhibited *Aeromonas* and *Vibrio* species *in vitro* and did not have any eukaryotic toxicity against zebra fish, bivalves, or mantis shrimp (50). The antibacterial activity of *Pseudoalteromonas* sp. JG1 was strongest against V. anguillarum *in vitro* (23), similar to what we observed in this study for *Pseudoalteromonas* sp. JC28. Several antibacterial compounds have been characterized from *Pseudoalteromonas* sp. JG1, including the Pfap protein and five small molecules: *p*-hydroxybenzoic acid, *trans*-cinnamic acid, 6-bromoindolyl-3-acetic acid, *N*-hydroxybenzoisoxazolone, and 2′-deoxydenosine, with the brominated compound (6-bromoindolyl-3-acetic acid) having the broadest antimicrobial activity (23). The Pfap protein is a putative l-amino acid oxidase, which may play a role in synthesis of 6-bromoindolyl-3-acetic acid (51). Future research will be needed to determine whether Pfap also contributes to the inhibitory activity of *Pseudoalteromonas* sp. JC28. Although *Pseudoalteromonas* sp. JG1 does contain a bromoalterochromide gene cluster (see Table S6 in the supplemental material), its activity has not been described.

Our efforts to chemically characterize *Pseudoalteromonas* sp. JC28 extracts led to the identification of the major bioactive components, the bromoalterochromides (BACs), including new derivatives. These brominated derivatives of alterochromides were first identified by matrix-assisted laser desorption/ionization mass spectrometry (MALDI-MS) from the yellow-pigmented, sponge tissue-derived P. maricaloris KMM636 (29), with full spectroscopic characterization of their structures published later (21), likely owing to low yields and compound instability. Relying on mass spectrometric methods, BACs have also been identified from several *Pseudoalteromonas* strains (22, 30, 52), indicating widespread metabolite expression within this bacterial genus. However, the complete structural elucidation of these compounds has been neglected, and inconsistencies in terms of their chemically determined and bioinformatically predicted absolute stereochemistry have been observed (19–21). However, in this study, through high-resolution nuclear magnetic resonance (NMR) spectroscopic and C_3_ Marfey’s techniques, we have identified the new derivatives, BAC-D/D′, and made the necessary corrections to the reported absolute stereochemistry of BAC-A/A′. The isolation of BAC-D/D′ with a d-*allo*-Ile in place of the d-Val of BAC-A/A′ is indicative of a biosynthetic flexibility that warrants further investigation.

The BACs produced by *Pseudoalteromonas* sp. JC28 demonstrated antibacterial activity against a Gram-positive bacterium and a Gram-negative bacterium and inhibited hyphal growth of the fungal pathogen *F. keratoplasticum.* However, there was a gap in antifungal activity (28% hyphal inhibition) of the purified compounds 1a/1b compared to that of the *Pseudoalteromonas* sp. JC28 bacterial strain or its extract when tested in a similar assay (96% and 61% hyphal inhibition, respectively [11]). A similar observation has been reported for other BAC-producing *Pseudoalteromonas* strains, which were initially active against *Vibrio anguillarium* in an isolate well diffusion assay, but whose ethyl acetate extracts were found to be inactive (52). These differences in activity could be caused by upregulation in BAC production during exposure to a pathogen, since other metabolites in the cell-free supernatant may be necessary to exert maximum hyphal growth inhibition or may be due to extraction protocols biasing toward organic-soluble metabolites. In another example, although BAC-A isolated from *Pseudoalteromonas* sp. strain J010 was previously shown to have antiprotozoal activity against Tetrahymena pyriformis, it did not have antibacterial or antifungal activity in a disc diffusion assay (22). Our study is the first demonstration that purified BACs themselves, devoid of the producer strain or other bacterial metabolites, possess antibacterial and antifungal activity, signifying the potential ecological importance of these PKS-NRPS molecules. Heterologous expression of the BAC gene cluster in E. coli (20) and optimization of *Pseudoalteromonas* culturing conditions (29) are promising methods to obtain higher yields of BACs for further biological investigations.

Besides potentially aiding in *E. scolopes* egg defense, the BACs may play a role in the ability of *Pseudoalteromonas* sp. JC28 to colonize the ANG. In the well-studied light organ symbiosis of *E. scolopes*, V. fischeri exclusively colonizes the light organ just a few hours after embryos hatch (53). Previous work has shown that reactive oxygen and nitrogen species (ROS and RNS) are important host components that help facilitate colonization (54–57) and V. fischeri has mechanisms to overcome NO stress (55, 58). The ability of BACs to inhibit NO production as reported herein may indicate a potential mechanism to overcome host defenses in the ANG, although this remains to be tested.

### Conclusions.

Here, we show that two strains associated with the squid ANG symbiosis demonstrated antimicrobial activity against other marine bacteria. One of these bioactive strains, *Pseudoalteromonas* sp. JC28, exhibited broad antimicrobial activity against both Gram-positive and Gram-negative bacteria, as well as fungi. The genome from this isolate represents the first sequenced member of the *Gammaproteobacteria* from the *E. scolopes* ANG symbiosis. Although pseudoalteromonads make up less than 0.1% of the ANG/JC bacterial community (12), it remains to be seen whether their strong bioactivity *in vitro* contributes to the overall defensive function in this symbiosis *in vivo*. We also show that *Pseudoalteromonas* sp. JC28 produces both known and new bromoalterochromides with antimicrobial and NO inhibitory effects, likely contributing to the bioactivity of this strain. Future research will focus on mutagenizing bromoalterochromide biosynthetic genes to determine the functional role of these compounds in the ANG symbiosis. Ongoing efforts to manipulate the ANG and egg communities (e.g., raising gnotobiotic squid with defined bacterial strains) will also allow for the characterization of antimicrobial metabolite production under *in vivo* conditions.

## MATERIALS AND METHODS

### Animal care statement.

All experimental procedures involving *E. scolopes* were conducted in accordance with protocols approved by the Institutional Animal Care & Use Committee (A18-029), Office of the Vice President for Research at the University of Connecticut and in compliance with the Office of Animal Welfare, National Institutes of Health and the Association for Assessment and Accreditation of Laboratory Animal Care International.

### Bacterial strains, identification, and culture conditions.

Fifteen of the bacterial strains used in this study were previously isolated from the accessory nidamental glands (ANGs) or jelly coats (JCs) of *E. scolopes* (see Table S1 in the supplemental material) (11, 13, 14). Four additional strains were isolated from ANGs of three squid as previously described (Table S1) (13). All strains were identified to the genus level by Sanger sequencing of the 16S rRNA gene as described previously (14). Sanger sequencing was performed by the Biotechnology Center at the University of Connecticut. All strains were grown on seawater tryptone (SWT) medium (5 g/liter tryptone, 3 g/liter yeast extract, 3 ml/liter glycerol, 700 ml/liter artificial seawater [Instant Ocean sea salts, Blacksburg, VA, USA], 15 g/liter agar, 300 ml/liter deionized [DI] water).

10.1128/mSphere.00166-20.4 TABLE S1 Microbial strains used in this study. Download Table S1, PDF file, 0.1 MB. Copyright © 2020 Suria et al. 2020 Suria et al. This content is distributed under the terms of the Creative Commons Attribution 4.0 International license.

### Zone of inhibition assay.

ANG and JC isolates were tested for their ability to inhibit marine Gram-positive bacteria (Bacillus megaterium CNJ 778 and Exiguobacterium aesteurii CNJ 771) and Gram-negative bacteria (Vibrio fischeri ES114, Vibrio anguillarum 775, Vibrio parahaemolyticus KNH1, Vibrio harveyi B392, and Photobacterium leiognathi KNH6) using an *in vitro* inhibition assay as previously described (14). Briefly, target strains were grown to stationary phase in 3 ml of SWT medium, shaking at 200 rpm, at 30°C and then spread onto SWT plates at a concentration of ∼10^8^ CFU/ml. The lawns were dried for 15 min, and then a stationary-phase culture of the ANG/JC isolate (∼10^8^ CFU/ml) was spotted (10 μl) onto the center of the plate. The plates were incubated at 28°C for 24 h. Due to the nonuniform morphology of the bacterial colonies, the zone of inhibition (ZOI) was defined here as the area (square centimeters), rather than diameter, around the ANG/JC isolate where no target bacteria grew (see Fig. S1 in the supplemental material for examples). This area was measured by subtracting the ANG/JC colony area from the ZOI using the image analysis program FIJI (60). A strain was marked as positive for inhibition if the ZOI was ≥0.15 cm^2^. Each ANG/JC isolate was tested against each marine target strain in experimental and technical triplicates.

10.1128/mSphere.00166-20.1 FIG S1 Representative images of zone of inhibition area measurements. Three examples of different interactions that were observed between ANG/JC isolates and target lawn strains. (A to C) A small ZOI area (0.16 cm^2^) around *Leisingera* sp. ANG59 against a V. fischeri lawn was observed. (D to F) A decrease in V. anguillarum target strain growth was observed around *Leisingera* sp. ANG7, but the small ZOI (0.11 cm^2^) was not marked as positive for inhibition. (G to I) A large ZOI (4.1 cm^2^) around *Pseudoalteromonas* sp. JC28 against V. fischeri was observed. Images in the first column (A, D, and G) depict the original plate image. Images in the second column (B, E, and H) feature the yellow region of interest line drawn in FIJI with the freehand selection tool to measure the area of the entire ZOI. Images in the third column (C, F, and I) feature the yellow region of interest line around the ANG/JC isolate to measure the colony area. All colony areas were subtracted from the entire ZOI areas to report the final ZOI. Images and measurements were taken as part of the initial ZOI screen summarized in Table 1. Scale bars, 1.0 cm (A to F) and 2.0 cm (G to I). Blue and black spots indicate orientation marks on the outside of the petri dish. Download FIG S1, PDF file, 0.8 MB. Copyright © 2020 Suria et al. 2020 Suria et al. This content is distributed under the terms of the Creative Commons Attribution 4.0 International license.

10.1128/mSphere.00166-20.2 FIG S2 NMR spectra of compounds 1a/1b in DMSO-*d_6_*. (a) ^1^H NMR spectrum of compounds 1a/1b in DMSO-*d_6_* (600 MHz); (b) ^1^H-^1^H COSY NMR spectrum of compounds 1a/1b in DMSO-*d_6_* (600 MHz); (c) HSQC NMR spectrum of compounds 1a/1b in DMSO-*d_6_* (600 MHz); (d) HMBC NMR spectrum of compounds 1a/1b in DMSO-*d_6_* (600 MHz); (e) NOESY NMR spectrum of compounds 1a/1b in DMSO-*d_6_* (600 MHz). Download FIG S2, PDF file, 1.2 MB. Copyright © 2020 Suria et al. 2020 Suria et al. This content is distributed under the terms of the Creative Commons Attribution 4.0 International license.

10.1128/mSphere.00166-20.3 FIG S3 NMR spectra of compounds 2a/2b in DMSO-*d_6_*. (a) ^1^H NMR spectrum of compounds 2a/2b in DMSO-*d_6_* (600 MHz); (b) ^1^H-^1^H COSY NMR spectrum of compounds 2a/2b in DMSO-*d_6_* (600 MHz); (c) HSQC NMR spectrum of compounds 2a/2b in DMSO-*d_6_* (600 MHz); (d) HMBC NMR spectrum of compounds 2a/2b in DMSO-*d_6_* (600 MHz); (e) NOESY NMR spectrum of compounds 2a/2b in DMSO-*d_6_* (600 MHz). Download FIG S3, PDF file, 0.9 MB. Copyright © 2020 Suria et al. 2020 Suria et al. This content is distributed under the terms of the Creative Commons Attribution 4.0 International license.

After the initial ZOI screen, the JC isolate, *Pseudoalteromonas* sp. JC28, was chosen for further testing based on its ability to inhibit all target strains. To test for density-dependent effects of this inhibitory activity, inhibition trials were retested with target strains at lower lawn densities of 10^4^, 10^5^, and 10^6^ CFU/ml as described above. Each test was performed in experimental and technical triplicates. For each target lawn strain, statistical significance between ZOIs at each lawn density was determined using one-way analyses of variance (ANOVAs). Multiple-comparison *post hoc* Tukey tests were performed to determine which lawn densities were significantly different.

### Genome sequencing.

*Leisingera* sp. ANG59 and *Pseudoalteromonas* sp. JC28 were grown in 3.0 ml of SWT broth, 200 rpm, at 30°C to a density of ∼10^8^ CFU/ml. Genomic DNA was extracted from 1.0 ml of culture using the MasterPure DNA Purification kit (Epicentre, Madison, WI, USA) following the manufacturer’s protocol. DNA quantity was measured with a high-sensitivity DNA Qubit fluorimeter (Invitrogen, Carlsbad, CA, USA), and quality was checked by absorbance measured on a Nanodrop spectrophotometer (Thermo Scientific, Waltham, MA, USA) and 1% agarose gel. DNA was prepped for sequencing using the Nextera XT DNA library prep kit (Illumina, San Diego, CA, USA). Library yield was checked with Qubit, and size and quality were measured on a high-sensitivity DNA bioanalyzer chip (Agilent Technologies, Santa Clara, CA, USA). Sequencing was performed on an Illumina MiSeq, reagent kit v.2 with 500 cycles at the Microbial Analysis, Resources, and Services (MARS) facility at the University of Connecticut.

Sequencing yielded 1,101,126 raw, paired-end reads for *Leisingera* sp. ANG59 and 3,976,074 raw, paired-end reads for *Pseudoalteromonas* sp. JC28, which were uploaded to the U.S. Department of Energy KBase server version 1.5.2 (https://kbase.us/) for analysis. Raw reads were trimmed and adaptors were removed using Trimmomatic (61). Reads were assembled using the A5 assembler (62). Assemblies were uploaded to the Rapid Annotation using Subsystem Technology (RAST) server (63) for annotations and to the antiSMASH server v.4.0 (64) for secondary metabolite biosynthetic gene cluster prediction. The *Leisingera* sp. ANG59 draft genome assembly has been deposited at DDBJ/ENA/GenBank under accession no. WLCM00000000 and is described in BioProject accession no. PRJNA589827. The *Pseudoalteromonas* sp. JC28 draft genome assembly has been deposited in DDBJ/EMBL/GenBank under accession no. QOKX00000000 and is described in BioProject accession no. PRJNA480278.

### Multilocus sequence analysis.

To determine the phylogenetic placement of *Pseudoalteromonas* sp. JC28 within the pseudoalteromonads, a multilocus sequence analysis (MLSA) tree was assembled using 34 published *Pseudoalteromonas* genomes. Six single-copy housekeeping genes were selected for multilocus sequence analysis based on a previous study (65), including the following: ATP synthase subunit beta, *atpD*; DNA gyrase subunit B, *gyrB*; rod shape-determining protein, *mreB*; RNA recombinase alpha subunit, *recA*; RNA polymerase sigma factor, *rpoD*; and DNA topoisomerase 1, *topA*. The genomes were downloaded from GenBank using a Python script (https://github.com/kblin/ncbi-genome-download). Amino acid sequences for each gene were obtained from each genome by a tblastn search using the amino acid sequence from E. coli strain K-12 obtained from UniProt. Extracted sequences were aligned using MUSCLE (66) with default parameters. The multiple alignments were concatenated in Geneious v.10 (Biomatters Inc., Newark, NJ, USA). The best evolutionary model of substitution was chosen by ModelFinder (67) in the IQtree webserver (68) as GTR + F + Invar + Gamma 4. A maximum likelihood tree was constructed using IQtree v.1.6.5 (69) with 1,000 bootstraps (70). The tree was visualized using the Interactive Tree of Life (iTOL) v.4 webserver (https://itol.embl.de/) (71). Studies indicating the pigmentation of a strain, featured on the MLSA tree (Fig. 2), are referenced in Table S2.

10.1128/mSphere.00166-20.5 TABLE S2 Pigmentation of *Pseudoalteromonas* strains included in MLSA tree (Fig. 2). Strains are listed in alphabetical order. Download Table S2, PDF file, 0.1 MB. Copyright © 2020 Suria et al. 2020 Suria et al. This content is distributed under the terms of the Creative Commons Attribution 4.0 International license.

10.1128/mSphere.00166-20.6 TABLE S3 Zone of inhibition experiments. (a) Zone of inhibition areas of ANG/JC isolates tested against seven marine bacteria *in vitro*. Areas (cm^2^) are averages ± standard errors of the means from three trials. (b) Zone of inhibition areas of *Pseudoalteromonas* sp. JC28 assayed against the target bacteria. Download Table S3, PDF file, 0.1 MB. Copyright © 2020 Suria et al. 2020 Suria et al. This content is distributed under the terms of the Creative Commons Attribution 4.0 International license.

10.1128/mSphere.00166-20.7 TABLE S4 Indigoidine biosynthetic genes detected in the *Leisingera* sp. ANG59 genome. Download Table S4, PDF file, 0.1 MB. Copyright © 2020 Suria et al. 2020 Suria et al. This content is distributed under the terms of the Creative Commons Attribution 4.0 International license.

10.1128/mSphere.00166-20.8 TABLE S5 Secondary metabolite biosynthetic gene clusters predicted by antiSMASH in the *Leisingera* sp. ANG59 and *Pseudoalteromonas* sp. JC28 genomes. Download Table S5, PDF file, 0.1 MB. Copyright © 2020 Suria et al. 2020 Suria et al. This content is distributed under the terms of the Creative Commons Attribution 4.0 International license.

10.1128/mSphere.00166-20.9 TABLE S6 Percent amino acid identity to bromoalterochromide gene cluster of BAC-containing *Pseudoalteromonas* genomes in MLSA tree (Fig. 2). Download Table S6, PDF file, 0.1 MB. Copyright © 2020 Suria et al. 2020 Suria et al. This content is distributed under the terms of the Creative Commons Attribution 4.0 International license.

10.1128/mSphere.00166-20.10 TABLE S7 Alterochromides detected by LC-MS in the EtOAc and 90% aqueous MeOH extracts of *Pseudoalteromonas* sp. JC28. Download Table S7, PDF file, 0.1 MB. Copyright © 2020 Suria et al. 2020 Suria et al. This content is distributed under the terms of the Creative Commons Attribution 4.0 International license.

The presence of the bromoalterochromide biosynthetic genes was determined by a tblastn search. The amino acid sequence for each bromoalterochromide (BGC0000314) biosynthetic gene was obtained from the Minimum Information about a Biosynthetic Gene cluster (MIBiG) server (72). This cluster was identified from Pseudoalteromonas piscicida JCM 20779 (19) and contains 14 genes (PpisJ2_010100002366 to PpisJ2_010100002431). A strain was marked as possessing the bromoalterochromide cluster if all 14 genes were present in the same cluster and had a high amino acid similarity (≥87%).

### Extraction of *Pseudoalteromonas* sp. JC28 and isolation of bromoalterochromides.

A preculture of *Pseudoalteromonas* sp. JC28 was prepared by inoculating the bacteria into 30 ml of marine broth (Difco Laboratories, Sparks, MD, USA) and incubating at 28°C for 24 h at 200 rpm. A 7.5-ml aliquot of the preculture was added into four 1-liter baffled flasks, each containing 500 ml of marine broth, before incubation at 28°C for 72 h at 200 rpm, protected from light. Solvents for extraction and semipreparative high-performance liquid chromatography (HPLC) purification were American Chemical Society (ACS) grade and HPLC grade, respectively, from Sigma-Aldrich (St. Louis, MO, USA). The extraction procedure was performed away from direct light exposure to prevent decomposition of the BACs (73).

Cultures were combined (2-liter total volume) and centrifuged at 6,300 rpm at 4°C for 10 min, and the supernatant was separated from the pellet via decantation. The supernatant was extracted with *n*-hexane (3 × 500 ml), followed by ethyl acetate (EtOAc) (12 × 300 ml) to afford 5.3 mg and 42.7 mg of extract, respectively. Meanwhile, the pellet was soaked in 100 ml of acetone overnight. The acetone extract was vacuum filtered and evaporated to dryness under reduced pressure before partitioning between *n*-hexane (200 ml) and 90% aqueous methanol (MeOH) (3 × 200 ml), to afford 7.8 mg and 69.7 mg of extract, respectively. Bromoalterochromides were detected by LC-MS analysis in the EtOAc supernatant extract, as well as in the MeOH pellet extract, using the following LC-MS conditions: column 150 × 4.6 mm Agilent Eclipse-XDB C_18_, 5 μm; column temperature of 24°C; flow rate of 0.7 ml/min; mobile phase A (0.1% HCOOH in water), mobile phase B ( 0.1% HCOOH in CH_3_CN), and a linear gradient of 40% mobile phase B to 100% mobile phase B over 30 min (Table S7). These two extracts were further purified by isocratic elution with 40% aqueous CH_3_CN at 2.0 ml/min using a semipreparative HPLC column (250 × 9.6 mm Agilent Eclipse XDB-C_18_ column, 5 μm), monitoring at 395 nm. The EtOAc extract yielded 0.8 mg of compounds 1a/1b and 0.4 mg of compounds 2a/2b, while the MeOH extract yielded 3.3 mg of compounds 1a/1b and 0.7 mg of compounds 2a/2b. Isolated compounds were stored in amber-colored vials under an inert atmosphere at −20°C.

### Characterization and confirmation of bromoalterochromide structures.

The planar structures of the bromoalterochromides (BACs) were determined by extensive nuclear magnetic resonance (NMR) analyses (^1^H, ^1^H-^1^H correlation spectroscopy [COSY], ^1^H-^13^C heteronuclear single quantum coherence [HSQC], ^1^H-^13^C heteronuclear multiple-bond correlation [HMBC], and nuclear Overhauser effect spectroscopy [NOESY]; Fig. S2 and S3) using a Varian INOVA 600-MHz instrument (Agilent Technologies, Santa Clara, CA, USA), equipped with a cryoprobe to enhance sensitivity. The NMR spectra were referenced to the solvent peaks of dimethyl sulfoxide DMSO-*d_6_* (δ_H_ = 2.50 ppm, δ_C_ = 39.52 ppm). High-resolution mass data were obtained using a Waters Xevo G2-XS QToF mass spectrometer (Waters Corporation, Milford, MA, USA), equipped with a ultraperformance liquid chromatography (UPLC) column (50 × 2.1 mm HSS T3 C_18_, 1.8 μm), and eluted using a gradient of mixtures of mobile phase A (0.1% aqueous HCOOH) and mobile phase B (0.1% HCOOH in CH_3_CN) beginning at 5% mobile phase B for 0.5 min, followed by a linear gradient from 5% mobile phase B to 60% mobile phase B for 3.5 min, then 60% mobile phase B to 98% mobile phase B for 4 min, and finally held at 98% mobile phase B for 1 min. The samples were dissolved in MeOH at 1 mg/ml, using 10-μl injections.

### Determination of absolute configuration for BAC-A/A′ and BAC-D/D′.

All standards and reagents were obtained from Sigma-Aldrich, except for dl-*allo*-Thr from Wako (Wako Chemicals USA, Inc., Richmond, VA, USA), and d-*allo*-Thr and 1-fluoro-2,4-dinitrophenyl-5-l-leucinamide (l-FDLA) from TCI (Tokyo Chemical Industry America, Portland, OR, USA). The LC-MS data were collected on an Agilent ESI single quadrupole mass spectrometer coupled to an Agilent 1260 HPLC system with a G1311 quaternary pump, G1322 degasser, and a G1315 diode array detector.

The absolute configuration of the BACs was determined by Marfey’s or advanced Marfey’s method as previously described (31, 74). The BAC 1a/1b or 2a/2b (200 μg) were heated at 105°C with 200 μl of 6 N HCl in a sealed flask for 3 to 4 h or until the reaction mixture turned colorless. The mixture was evaporated to dryness under nitrogen, and the resulting residue was dissolved in 50 μl of water and 20 μl of 1 M NaHCO_3_. Then, 40 μl of 1% l-FDLA or 1% 1-fluoro-2,4-dinitrophenyl-5-l-alaninamide (l-FDAA) in acetone was added prior to incubation at 35°C for 1 h. The reaction was quenched by the addition of 20 μl of 1 N HCl, then diluted with 370 μl of MeOH, from which 25 μl was injected for LC-MS analysis. The alkalinified solutions of the l and d forms of standard amino acids (100 μg), Val, Leu, Ile, Thr, Asn, and Asp, were individually derivatized in the same manner as the natural products. After quenching with 20 μl of 1 N HCl, they were diluted with 810 μl of MeOH, from which 25 μl was injected for LC-MS analysis. LC-MS conditions were as follows for FDLA-derived samples: column 150 × 4.6 mm Agilent Zorbax-SB C_3_, 5 μm; column temperature, 40°C; flow rate, 0.7 ml/min; mobile phase A is 5% CH_3_CN with 1% HCOOH in water and mobile phase B is 5% CH_3_CN with 1% HCOOH in MeOH; and linear gradient of 30% mobile phase B to 100% mobile phase B over 55 min. LC-MS conditions were as follows for FDAA-derived samples: column 150 × 4.6 mm Agilent Zorbax-SB C_3_, 5 μm; column temperature, 25°C; flow rate, 0.7 ml/min; mobile phase A is 7.5% CH_3_CN with 1% HCOOH in water and mobile phase B is 7.5% CH_3_CN with 1% HCOOH in MeOH; and linear gradient of 12.5% mobile phase B to 57.5% mobile phase B over 80 min.

### 96-well antibacterial assay.

The BACs (compounds 1a/1b and compounds 2a/2b) were tested against the Gram-positive bacterium Bacillus algicola CNJ 803 and the Gram-negative bacterium V. fischeri ES114 in 96-well liquid assays, following previously described methodology (14). Prior to the assays, *B. algicola* was grown on nutrient agar (BD, Franklin Lakes, NJ, USA) and V. fischeri on SWT agar, overnight at 28°C. Inocula of the target strains were prepared by adding colonies to nutrient broth or SWT broth and adjusting the optical density at 600 nm (OD_600_) to 0.08 to 0.10. A master mix was then prepared by mixing 7.84 ml of the appropriate broth, 6.40 ml water, and 1.60 ml of the inoculum. Into a 96-well round-bottomed plate (Corning Costar, Corning, NY, USA), 199 μl of the master mix was added with 1 μl of sample or controls. The final testing concentrations were 250 μg/ml for extracts and 0.39 to 50 μg/ml for purified compounds, dissolved in dimethyl sulfoxide (DMSO) (0.5% final concentration). The positive controls were 2.5 μg/ml of vancomycin (Sigma-Aldrich, St. Louis, MO) for *B. algicola* and 2.5 μg/ml of chloramphenicol (Acros Organics, Morris, NJ, USA) for V. fischeri, while the negative control was DMSO. The plate was incubated for 24 h at 28°C and with constant shaking at 200 rpm in the case of V. fischeri. The wells were observed visually for the presence or absence of bacterial growth to determine the MIC. The assays were done in technical triplicates with at least two experimental replicates.

### Nitric oxide inhibition assay.

Compounds 1a/1b were tested for inhibition of nitric oxide (NO) using previously described methods (75). Briefly, aliquots (197 μl) of RAW264.7 macrophage cells in Dulbecco modified Eagle medium (DMEM), high glucose, pyruvate (Thermo Fisher Scientific), supplemented with 10% fetal bovine serum (Thermo Fisher Scientific) and 1% penicillin-streptomycin (Sigma-Aldrich), were seeded into a 96-well flat-bottomed plate (Corning Costar) at a density of 2.5 × 10^4^ cells/well, and incubated at 37°C in an atmosphere of 5% CO_2_. After 24 h, 2 μl of LPS (final concentration, 100 ng/ml) and 1 μl of compounds 1a/1b (dissolved in DMSO; final concentrations between 1.56 and 50 μg/ml) or controls (10 μM suberoylanilide hydroxamic acid [SAHA] [Sigma-Aldrich] as a positive control; DMSO as a negative control) was added to the well and incubated for an additional 24 h. Then, 100 μl of the supernatant was transferred into another flat-bottomed 96-well plate, to which 50 μl of 1% sulfanilamide (Sigma-Aldrich) in 5% phosphoric acid (Aqua Solutions, Inc., Deer Park, TX, USA) was added. After 5 min, 50 μl of 0.1% aqueous *N*-(1-naphthyl)-ethylenediamine (NED) was mixed into the wells. The plates were read after another 5 min at 540 nm using a Synergy H1 hybrid reader (Biotek, Winooski, VT, USA). NO production was expressed as a percentage of the ratio between the sample absorbance and that of the negative control, DMSO. The assay was done in technical triplicates with two experimental replicates.

### Antifungal disc assay.

An antifungal assay was performed using previously described methodology with slight modification (76). Fusarium keratoplasticum FSSC-2g was spread onto an SWT agar plate and allowed to grow at 28°C for 24 to 48 h. An inoculum with an OD_600_ of 0.01 was prepared in SWT broth from F. keratoplasticum colonies on the plate. Sterile 6-mm paper discs (Difco Laboratories) were placed on top of SWT agar plates, to which 10 μl of sample or controls was added. Compounds 1a/1b, dissolved in DMSO, was tested at 10 μg to 100 μg/disc, with cycloheximide (Sigma-Aldrich) at 68 μg/disc as a positive control and 10 μl of DMSO as a negative control. Discs were allowed to dry for 5 min before adding the fungal inoculum onto the discs. The plates were then incubated at 28°C and observed for hyphal growth every 24 h for 3 days. Activity was measured by reduction in area of hyphal growth of the sample relative to the negative control.

### Data availability.

The *Pseudoalteromonas* sp. JC28 draft genome assembly has been deposited in DDBJ/EMBL/GenBank under accession no. QOKX00000000 and is described in BioProject PRJNA480278. The version described in this paper is version QOKX01000000. The *Leisingera* sp. ANG59 draft genome assembly has been deposited at DDBJ/ENA/GenBank under the accession no. WLCM00000000 and is described in BioProject PRJNA589827. The version described in this paper is version WLCM01000000.
