# Supplementary material for: Hawaiian Bobtail Squid Symbionts Inhibit Marine Bacteria via Production of Specialized Metabolites, Including New Bromoalterochromides BAC-D/D′
Source: mSphere. 2020 Jul 1;5(4):e00166-20. doi: 10.1128/mSphere.00166-20 (PMC7333567; doi:10.1128/mSphere.00166-20)
Supplement: TABLE S1 [file mSphere.00166-20-st001.pdf]

Table S1. Microbial strains used in this study

Strain	Characteristics	Source
Alteromonas sp. JC21	Isolate from *E. scolopes* egg jelly coats	Kerwin et al. (2019)
Bacillus algicola CNJ 803	Marine sediment sample, 0meter depth, Republic of Palau	Gontang et al. (2007) (Courtesy of Dr. René Augustin and the McFall-Ngai/Ruby Labs, University of Hawaii)
Bacillus megaterium CNJ 778	Marine sediment sample, 23meter depth, Republic of Palau	Gontang et al. (2007) (Courtesy of Dr. René Augustin and the McFall-Ngai/Ruby Labs, University of Hawaii)
Exiguobacterium aestuarii CNJ 771	Marine sediment sample, 50meter depth, Republic of Palau	Gontang et al. (2007) (Courtesy of Dr. René Augustin and the McFall-Ngai/Ruby Labs, University of Hawaii)
Fusarium keratoplasticum FSSC-2g	Isolate from fouling antibiotic-treated *E. scolopes* eggs	Kerwin et al. (2019)
Labrenzia sp. ANG18	Isolate from *E. scolopes* ANG	Kerwin et al. (2019)
Leisingera sp. ANG1	Isolate from *E. scolopes* ANG	Collins & Nyholm (2011)
Leisingera sp. ANG7	Isolate from *E. scolopes* ANG	This study
Leisingera sp. ANG13	Isolate from *E. scolopes* ANG	Kerwin et al. (2019)
Leisingera sp. ANG52	Isolate from *E. scolopes* ANG	This study
Leisingera sp. ANG59	Isolate from *E. scolopes* ANG	This study
Leisingera sp. ANG-DT	Isolate from *E. scolopes* ANG	Collins et al. (2015)
Leisingera sp. ANG-M6	Isolate from *E. scolopes* ANG	Collins et al. (2015)
Leisingera sp. ANG-S	Isolate from *E. scolopes* ANG	Collins et al. (2015)
Leisingera sp. ANG-S3	Isolate from *E. scolopes* ANG	Collins et al. (2015)
Leisingera sp. JC11	Isolate from *E. scolopes* egg jelly coats	Kerwin et al. (2019)
Muricauda sp. ANG21	Isolate from *E. scolopes* ANG	Gromek et al. (2016)
Photobacterium leiognathi KNH6	Seawater isolate	Stabb & Ruby (2002)
Pseudoalteromonas sp. JC28	Isolate from *E. scolopes* egg jelly coats	Kerwin et al. (2019)
Ruegeria sp. ANG6	Isolate from *E. scolopes* ANG	Kerwin et al. (2019)
Ruegeria sp. ANG10	Isolate from *E. scolopes* ANG	Kerwin et al. (2019)
Ruegeria sp. ANG-S4	Isolate from *E. scolopes* ANG	Collins et al. (2015)
Shewanella sp. ANG44	Isolate from *E. scolopes* ANG	This study
Vibrio anguillarum 775	Isolate from *Oncorhynchus kisutch*	Crosa et al. (1977) (Courtesy of Dr. Joerg Graf, University of Connecticut)

Vibrio fischeri ES114	Isolate from *E. scolopes* light organ	Boettcher & Ruby (1990)
Vibrio harveyi B392	Seawater isolate	Reichelt & Baumann (1973)
Vibrio sp. JC34	Isolate from *E. scolopes* egg jelly coats	Kerwin et al. (2019)

References

Boettcher, K. J., & Ruby, E. G. (1990). Depressed light emission by symbiotic by Vibrio fischerie of the sepiolid squid Euprymma scolopes. *J. Bacteriol.*, *172*(7), 3701–3706.

Collins, A. J., Fullmer, M. S., Gogarten, J. P., & Nyholm, S. V. (2015). Comparative genomics of Roseobacter clade bacteria isolated from the accessory nidamental gland of *Euprymna scolopes*. *Frontiers in Microbiology*, *6*(February), 1–14. https://doi.org/10.3389/fmicb.2015.00123

Collins, A. J., & Nyholm, S. V. (2011). Draft genome of *Phaeobacter gallaeciensis* ANG1, a dominant member of the accessory nidamental gland of *Euprymna scolopes*. *Journal of Bacteriology*, *193*(13), 3397–3398. https://doi.org/10.1128/JB.05139-11

Crosa, J. H., Schiewe, M. H., & Falkow, S. (1977). Evidence for plasmid contribution to the virulence of the fish pathogen Vibrio anguillarum. *Infection and Immunity*, *18*(2), 509–513.

Gontang, E. A., Fenical, W., & Jensen, P. R. (2007). Phylogenetic Diversity of Gram-Positive Bacteria Cultured from Marine Sediments □ †, *73*(10), 3272–3282. https://doi.org/10.1128/AEM.02811-06

Gromek, S. M., Suria, A., Fullmer, M. S., Garcia, J. L., Gogarten, J. P., Nyholm, S. V, & Balunas, M. J. (2016). *Leisingera* sp. JC1, a bacterial isolate from Hawaiian bobtail squid eggs, produces indigoidine and differentially inhibits vibrios. *Frontiers in Microbiology*, *7*(September). https://doi.org/10.3389/fmicb.2016.01342

Kerwin, A. H., Gromek, S. M., Suria, A. M., Samples, R. M., Deoss, D. J., O'Donnell, K., … Nyholm, S. V. (2019). Shielding the Next Generation: Symbiotic Bacteria from a Reproductive Organ Protect Bobtail Squid Eggs from Fungal Fouling. *MBio*, *10*(5), 1–18. https://doi.org/10.1128/mBio.02376-19

Reichelt, J. L., & Baumann, P. (1973). Taxonomy of the marine, luminous bacteria. *Archiv Fur Mikrobiologie*, *94*(4), 283–330.

Stabb, E. V., & Ruby, E. G. (2002). RP4-based plasmids for conjugation between Escherichia coli and members of the vibrionaceae. *Methods in Enzymology*, *358*(1997), 413–426. https://doi.org/10.1016/S0076-6879(02)58106-4
