# Supplementary figures and images for: Hawaiian Bobtail Squid Symbionts Inhibit Marine Bacteria via Production of Specialized Metabolites, Including New Bromoalterochromides BAC-D/D′
Source: mSphere. 2020 Jul 1;5(4):e00166-20. doi: 10.1128/mSphere.00166-20 (PMC7333567; doi:10.1128/mSphere.00166-20)

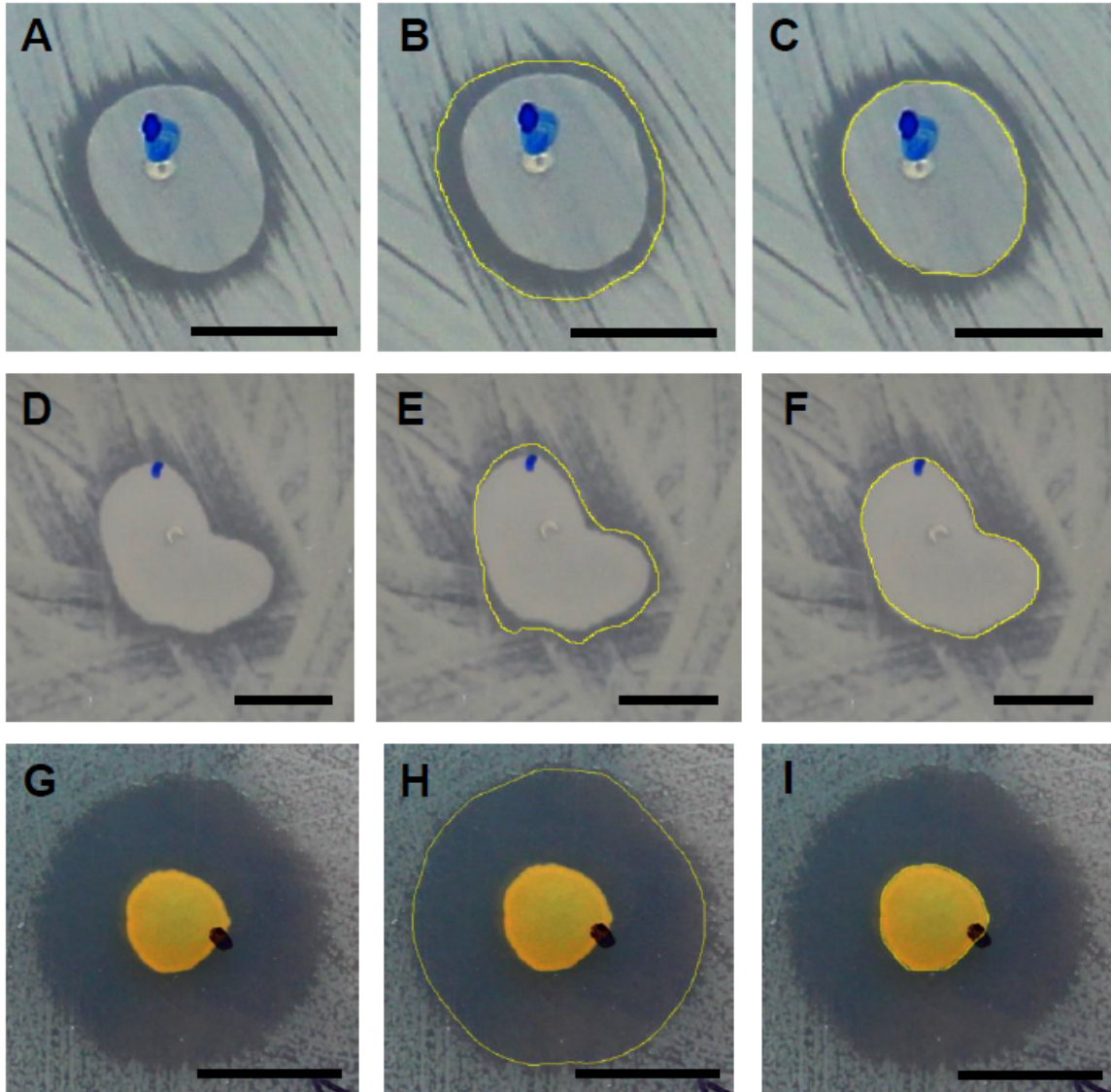

**Figure S1. Representative images of zone of inhibition area measurements.**

Supplement: FIG S1 [file mSphere.00166-20-sf001.pdf]
