# Supplementary material for: Hawaiian Bobtail Squid Symbionts Inhibit Marine Bacteria via Production of Specialized Metabolites, Including New Bromoalterochromides BAC-D/D′
Source: mSphere. 2020 Jul 1;5(4):e00166-20. doi: 10.1128/mSphere.00166-20 (PMC7333567; doi:10.1128/mSphere.00166-20)
Supplement: FIG S2 [file mSphere.00166-20-sf002.pdf]

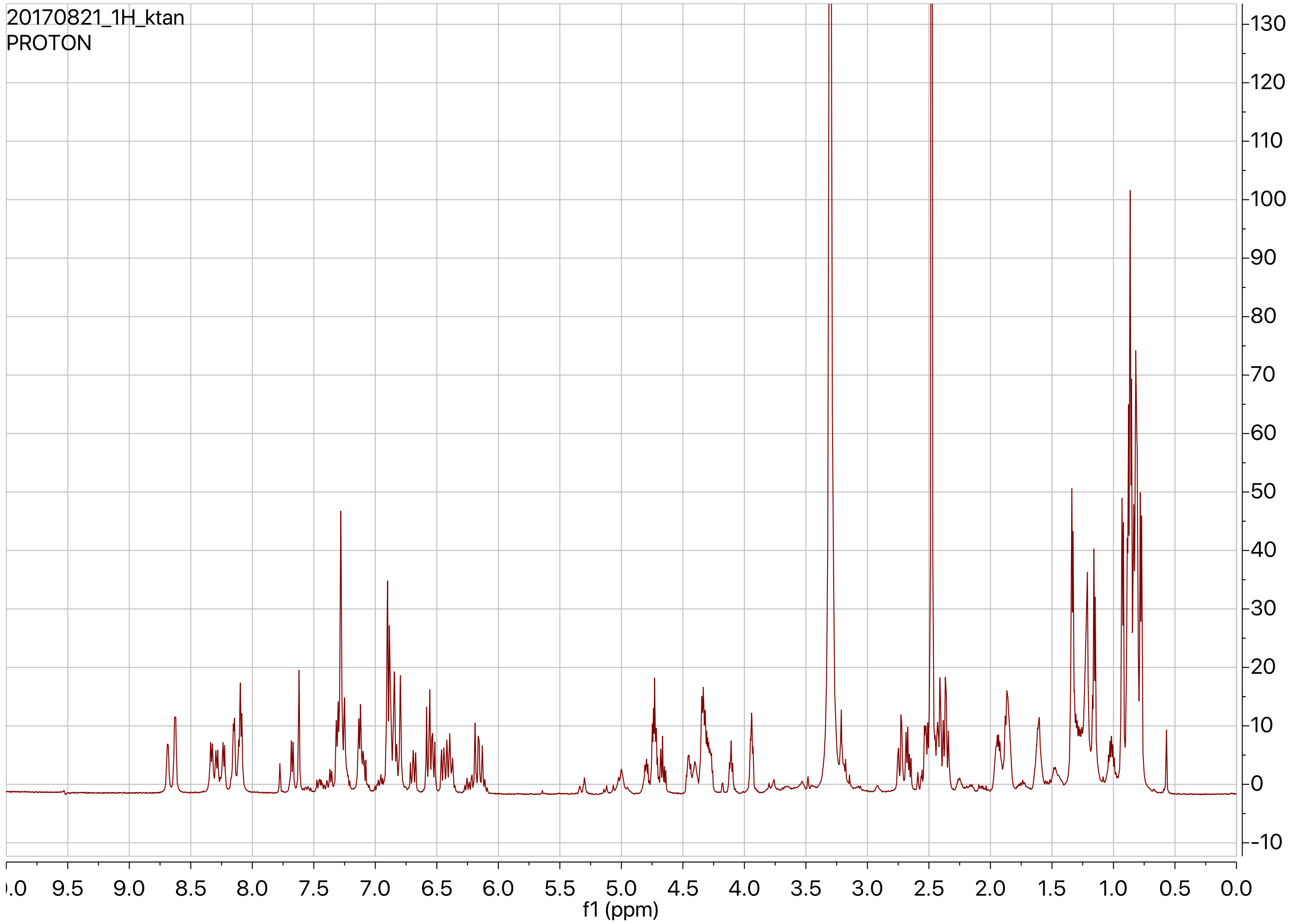

Figure S2a. ^{1}H NMR spectrum of **1a**/**1b** in DMSO-d_6 (600 MHz).

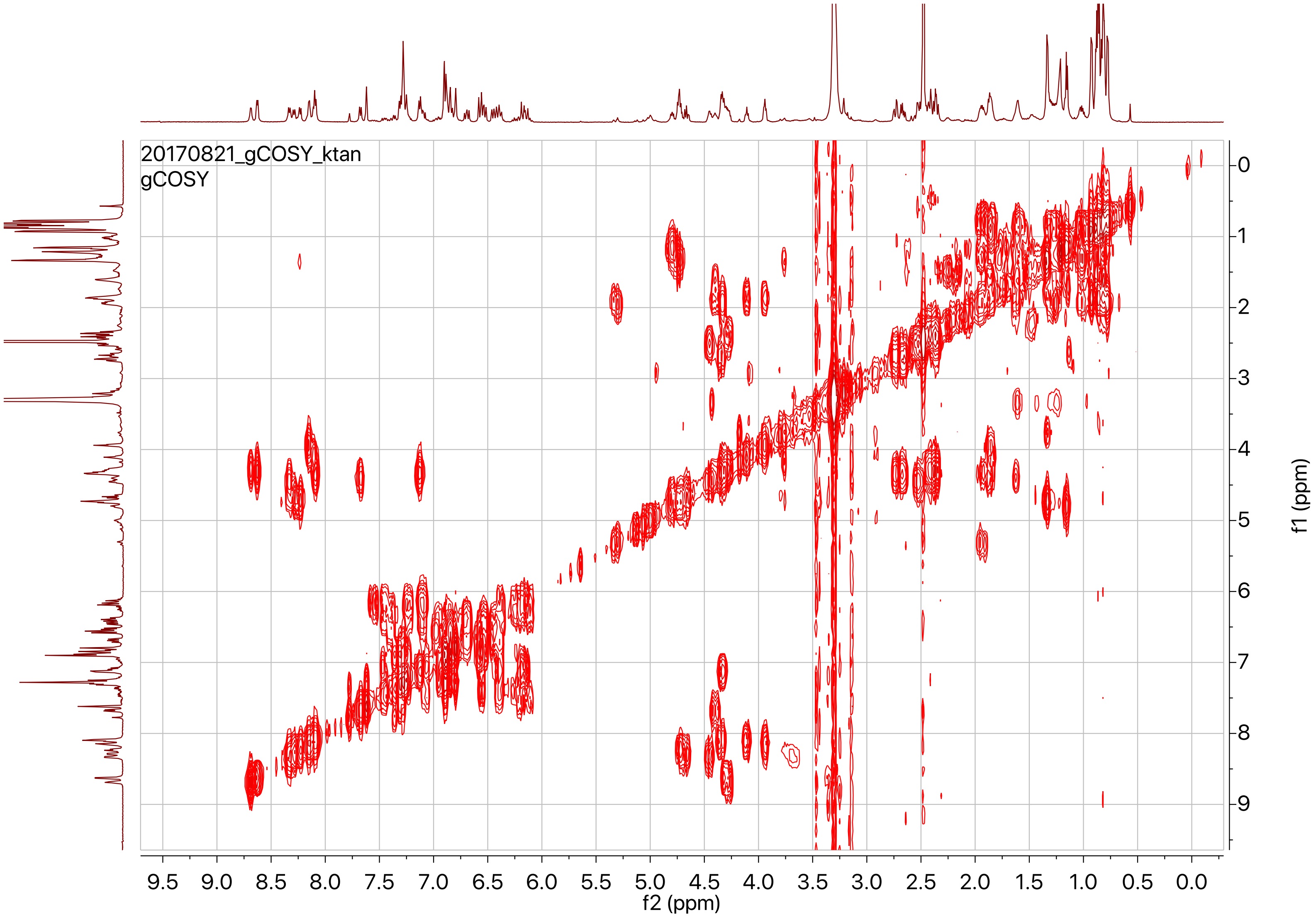

Figure S2b. ^{1}H-^{1}H COSY NMR spectrum of **1a**/**1b** in DMSO-d_6 (600 MHz).

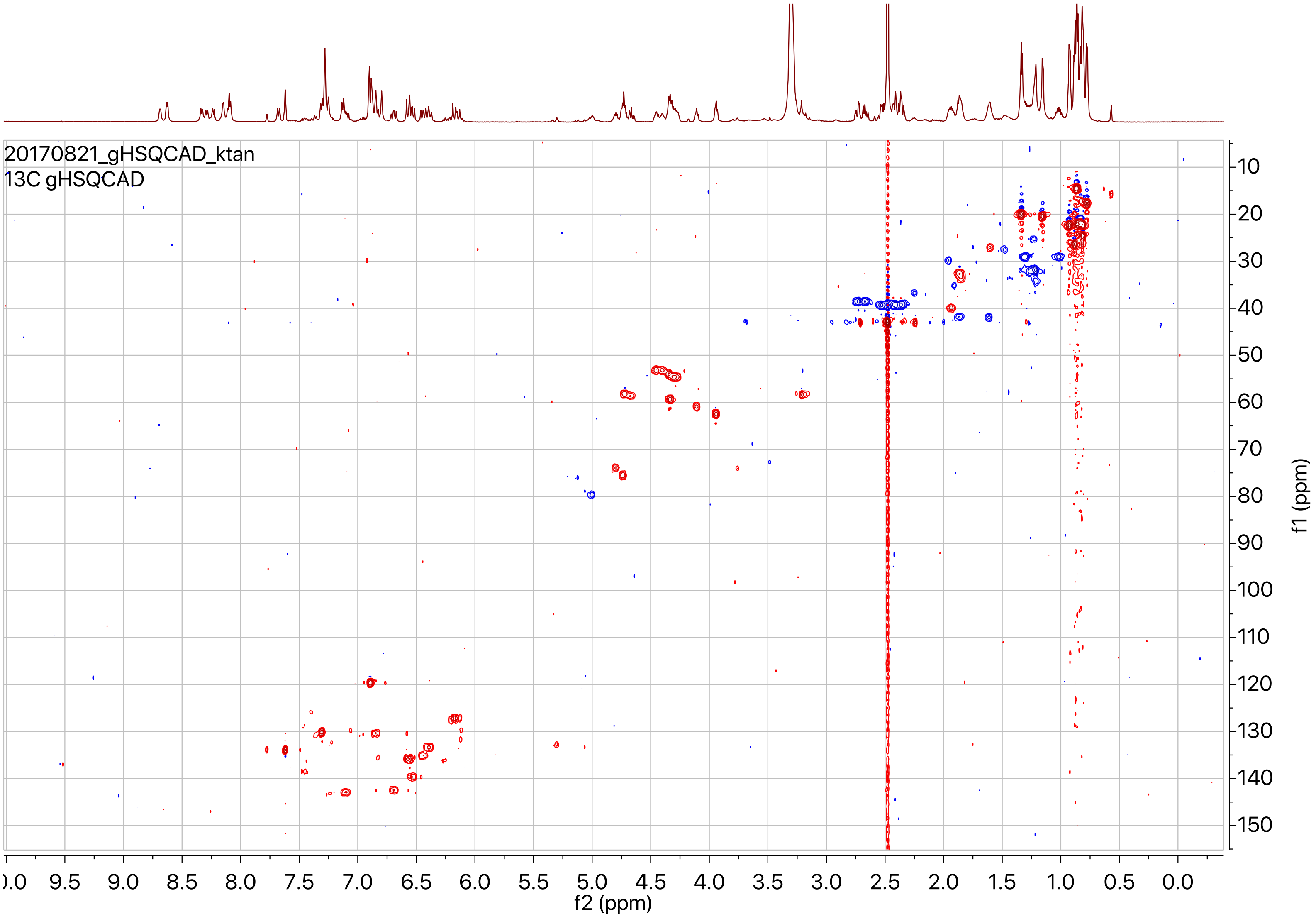

Figure S2c. HSQC NMR spectrum of **1a**/**1b** in DMSO-d_6 (600 MHz).

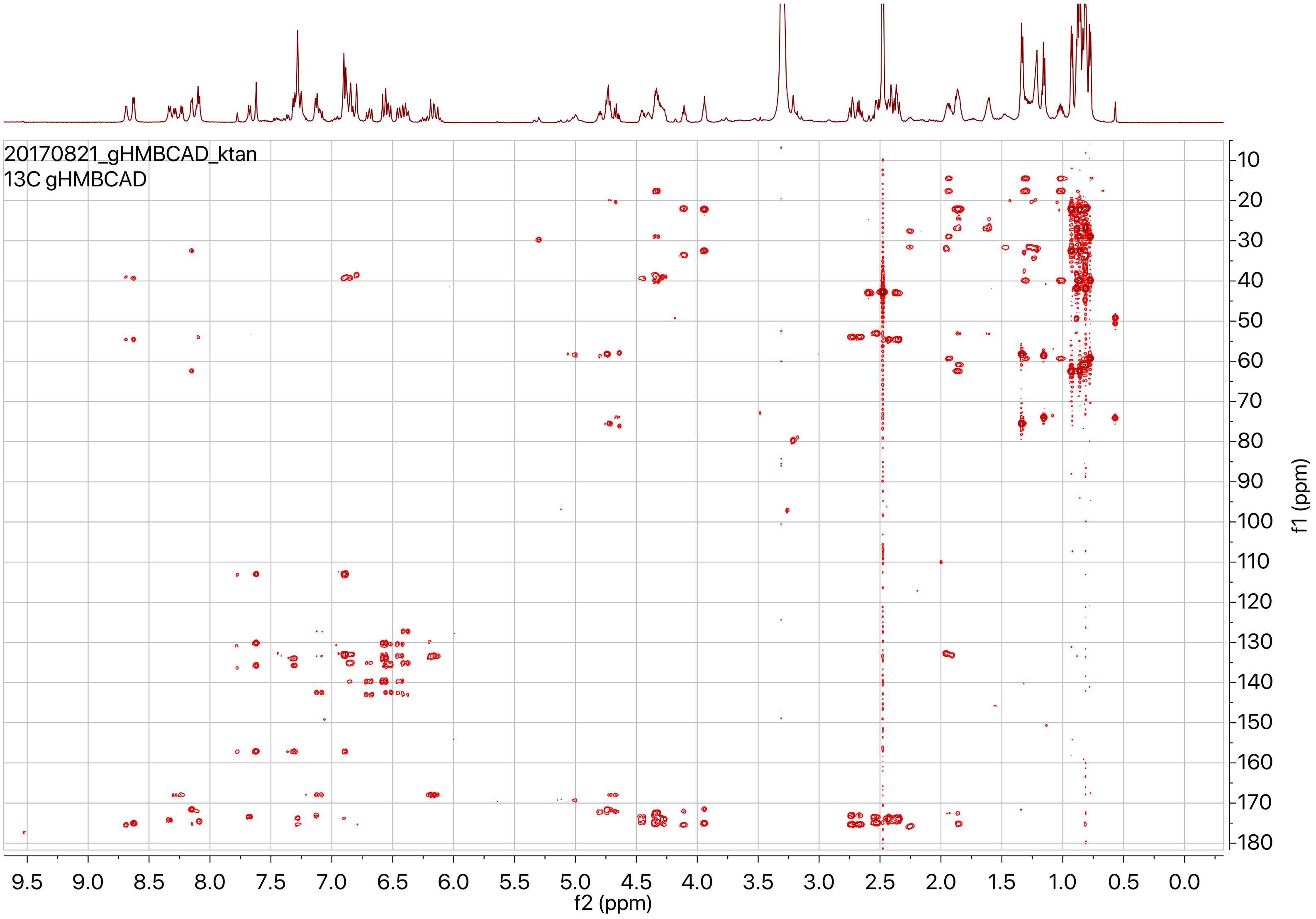

Figure S2d. HMBC NMR spectrum of **1a**/**1b** in DMSO-d_6 (600 MHz).

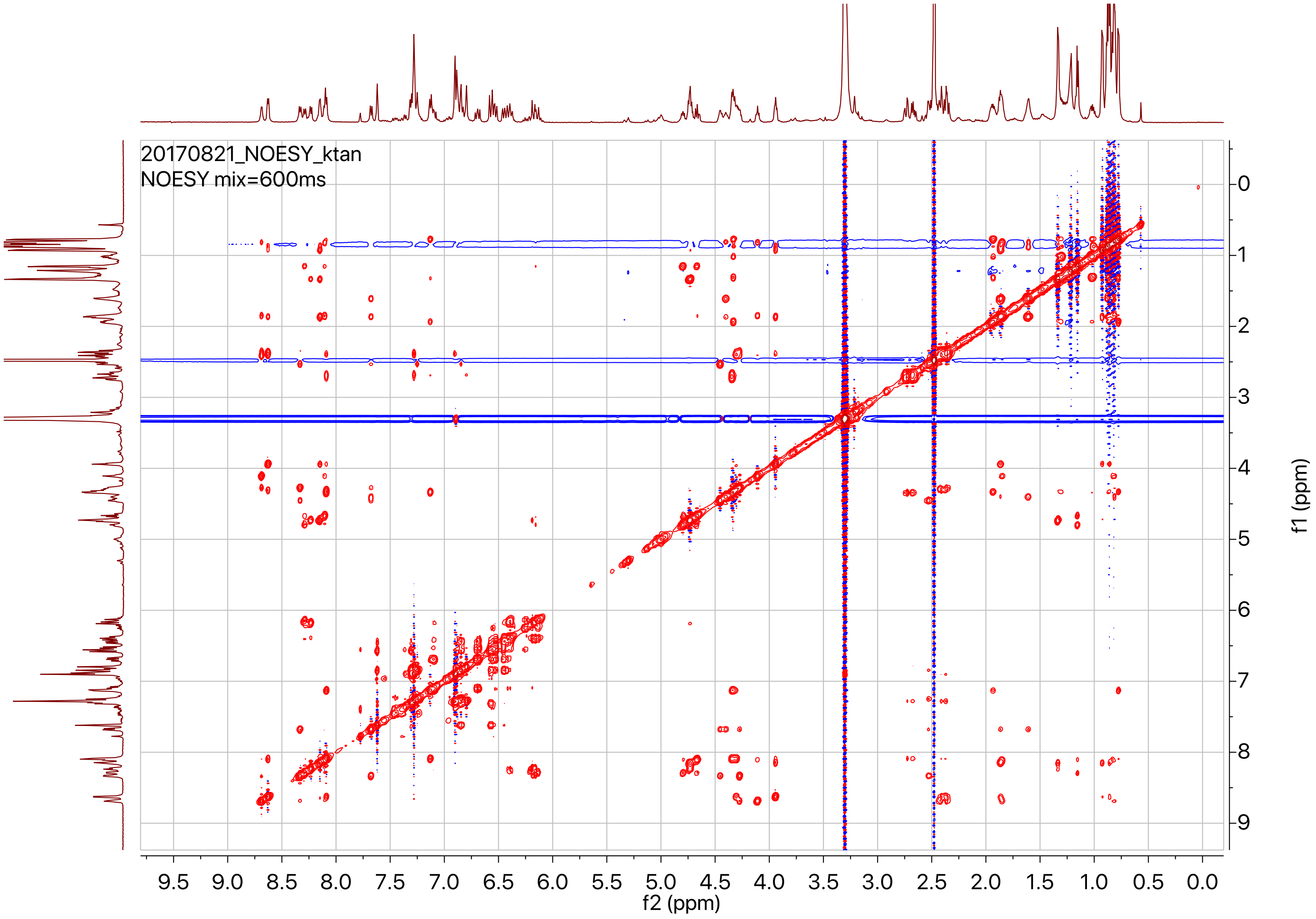

Figure S2e. NOESY NMR spectrum of **1a**/**1b** in DMSO-d_6 (600 MHz).
