# Supplementary material for: Hawaiian Bobtail Squid Symbionts Inhibit Marine Bacteria via Production of Specialized Metabolites, Including New Bromoalterochromides BAC-D/D′
Source: mSphere. 2020 Jul 1;5(4):e00166-20. doi: 10.1128/mSphere.00166-20 (PMC7333567; doi:10.1128/mSphere.00166-20)
Supplement: FIG S3 [file mSphere.00166-20-sf003.pdf]

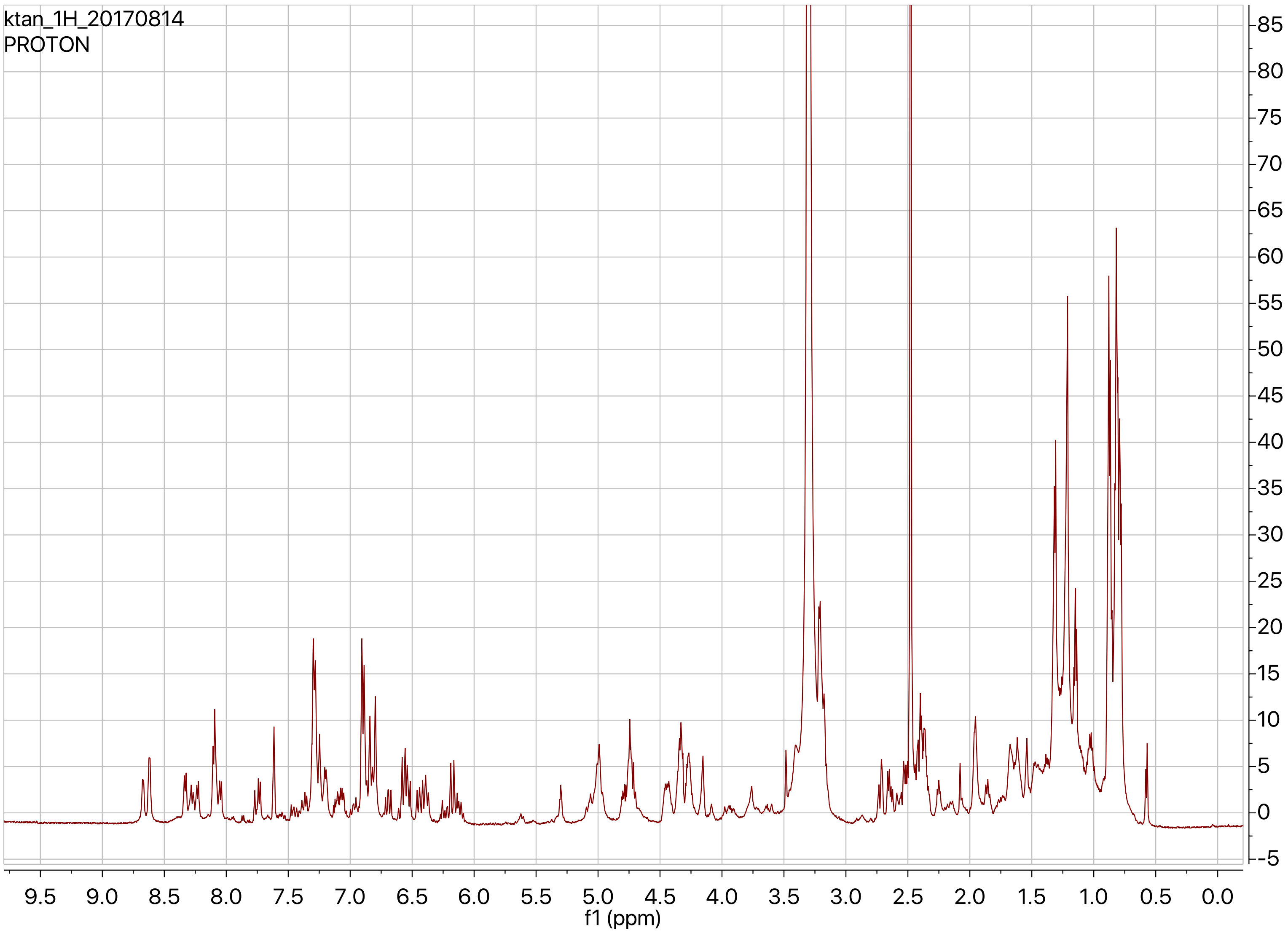

Figure S3a. ^{1}H NMR spectrum of **2a**/**2b** in DMSO-d_6 (600 MHz).

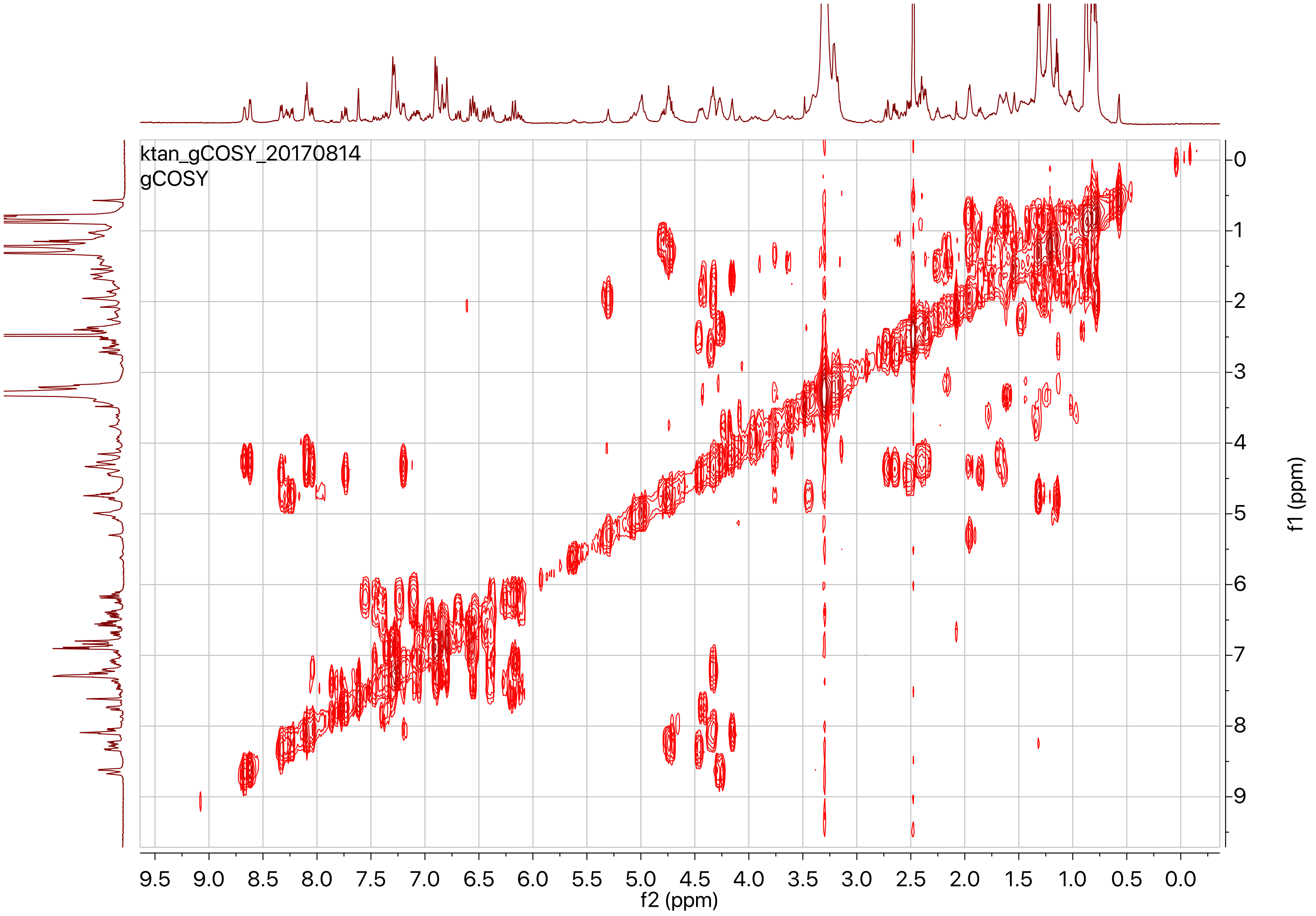

Figure S3b. ^{1}H-^{1}H COSY NMR spectrum of **2a**/**2b** in DMSO-d_6 (600 MHz).

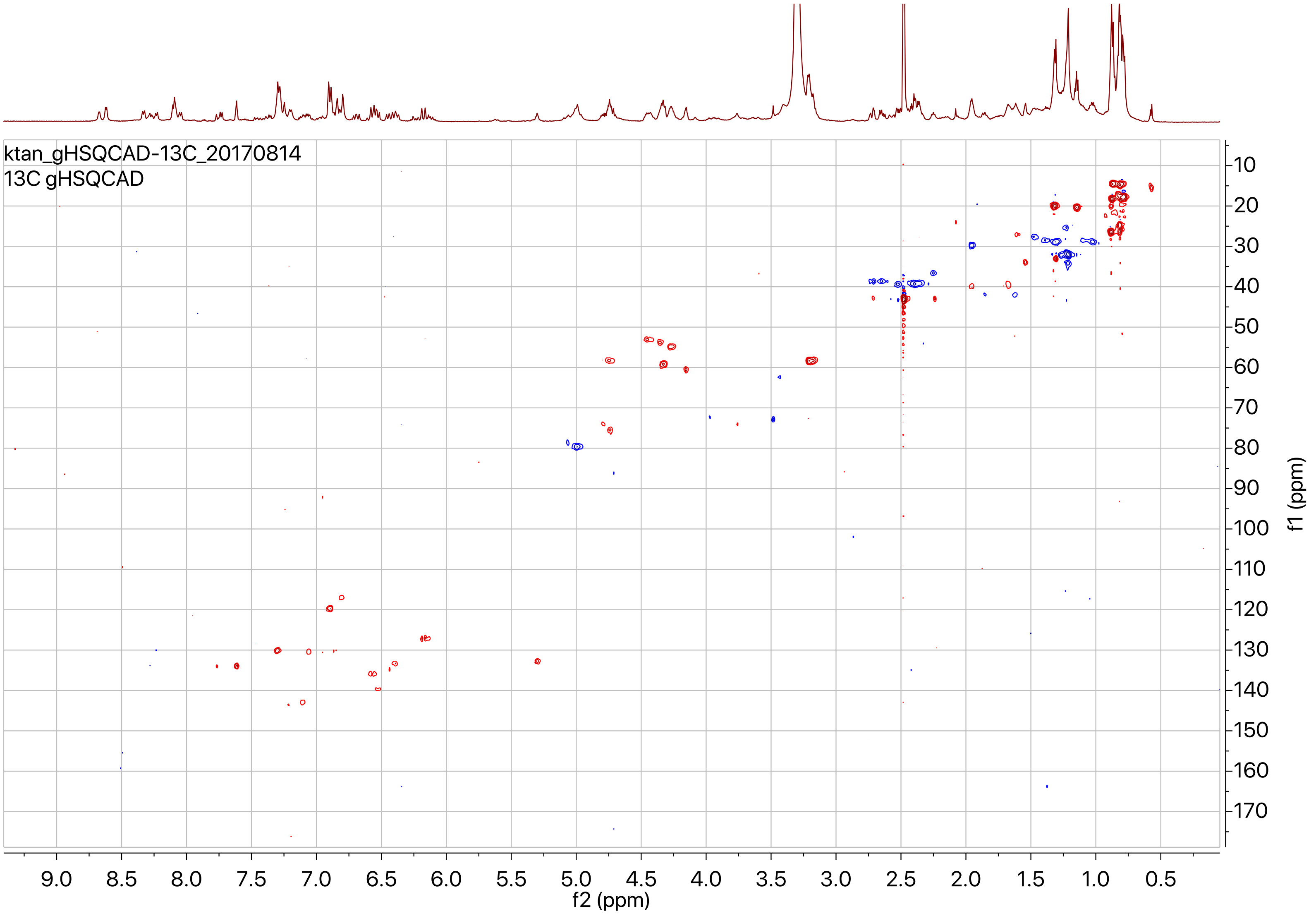

Figure S3c. HSQC NMR spectrum of **2a**/**2b** in DMSO-d_6 (600 MHz).

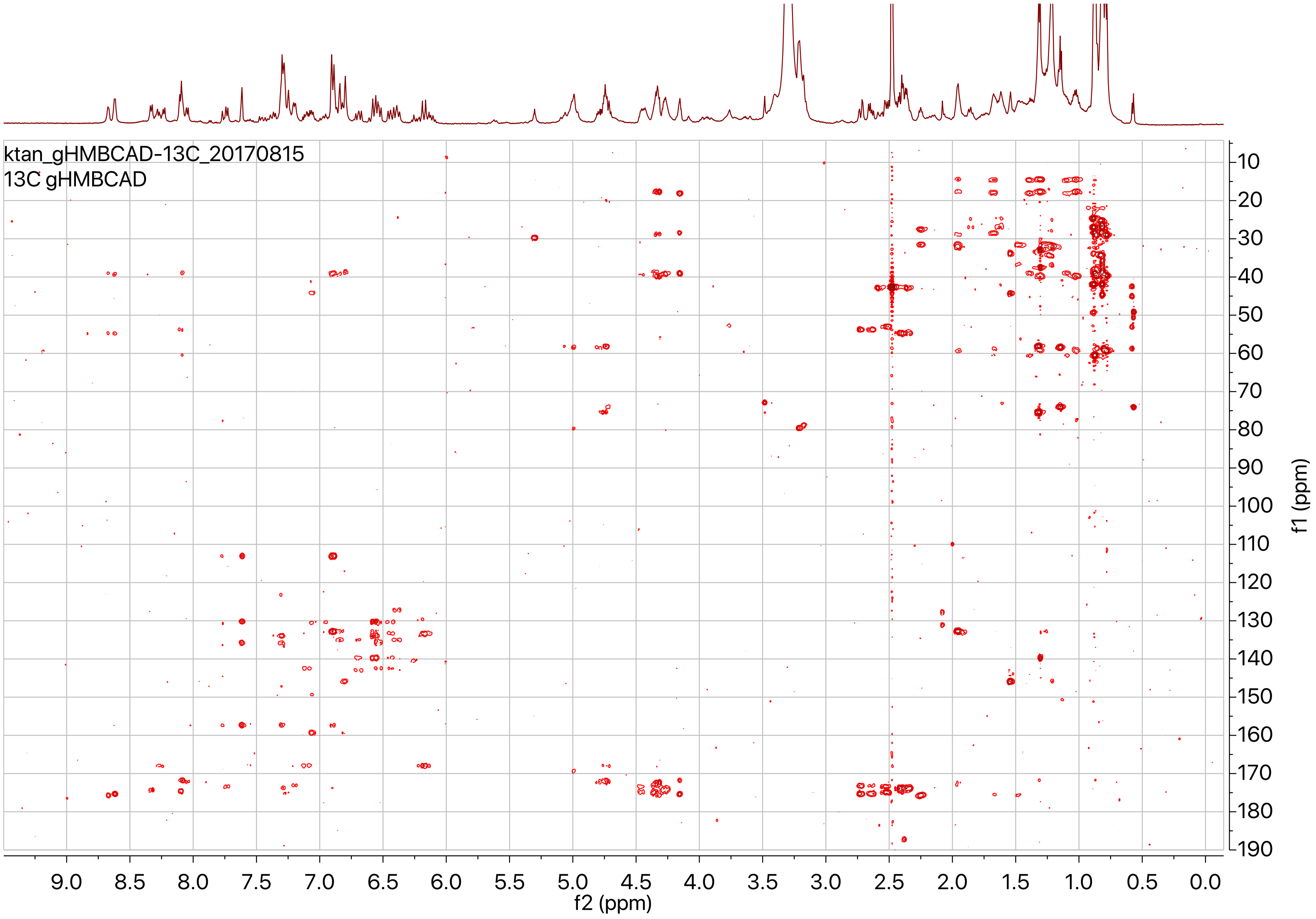

Figure S3d. HMBC NMR spectrum of **2a**/**2b** in DMSO-d_6 (600 MHz).

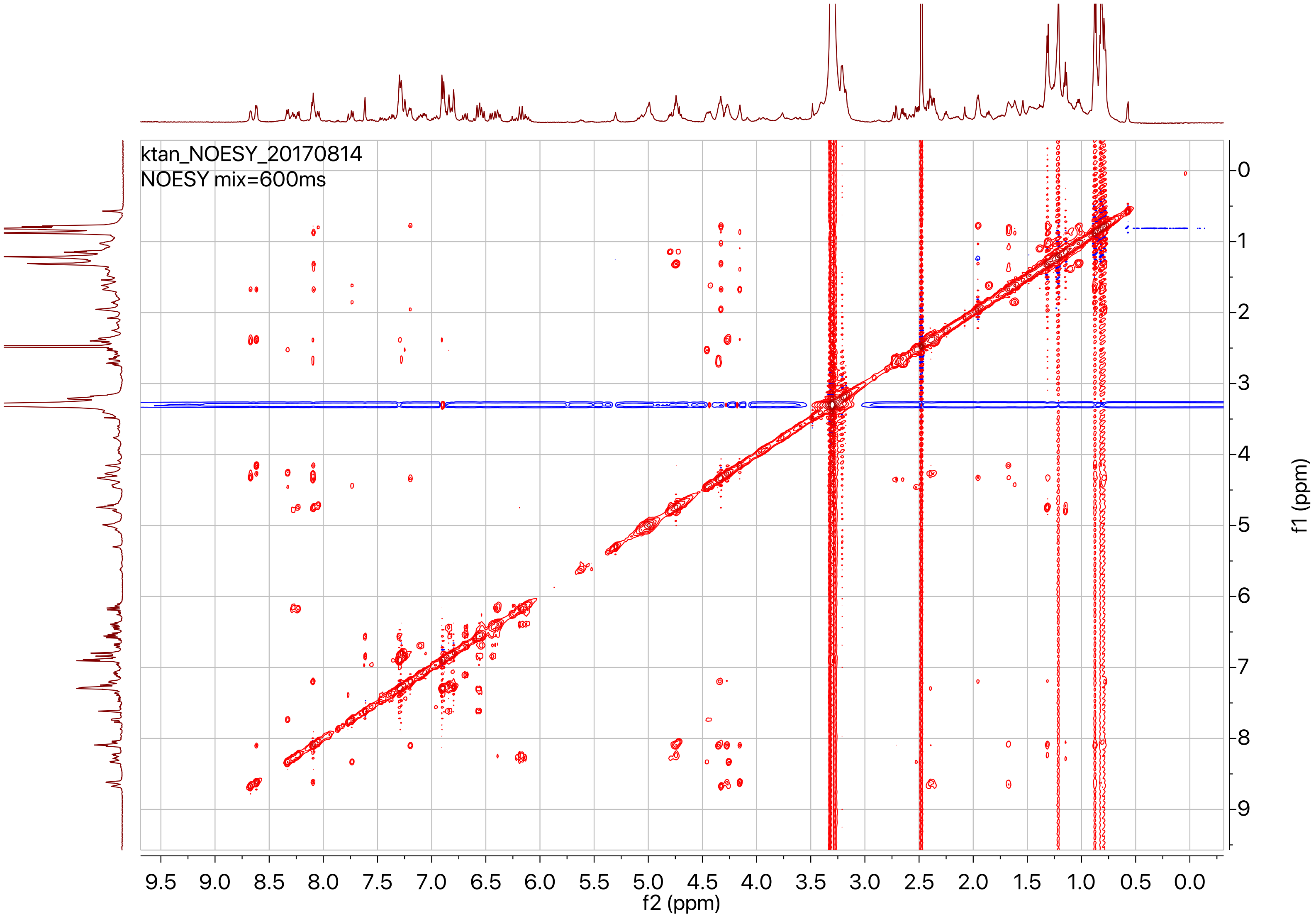

Figure S3e. NOESY NMR spectrum of **2a**/**2b** in DMSO-d_6 (600 MHz)
