# Supplementary material for: Hawaiian Bobtail Squid Symbionts Inhibit Marine Bacteria via Production of Specialized Metabolites, Including New Bromoalterochromides BAC-D/D′
Source: mSphere. 2020 Jul 1;5(4):e00166-20. doi: 10.1128/mSphere.00166-20 (PMC7333567; doi:10.1128/mSphere.00166-20)
Supplement: TABLE S2 [file mSphere.00166-20-st002.pdf]

**Table S2.** Pigmentation of *Pseudoalteromonas* strains included in MLSA tree (Fig. 2). Strains listed in alphabetical order

| **Strain** | **Pigmentation** | **Reference** |
|---|---|---|
| *P. aliena* EH1 | No | (Bernbom, Ng, Kjelleberg, Harder, & Gram, 2011) |
| *P. agarivorans* S816 | No | (Romanenko, Zhukova, Rohde, et al., 2003) |
| *P. arabiensis* JCM17292 | No | (Matsuyama et al., 2013) |
| *P. arctica* A3712 | No | (Xie et al., 2012) |
| *P. byunsanensis* JCM12483 | Yes | (Park, Bak, Yi, Bae, & Chun, 2005) |
| *P. citrea* NCIMB1889 | Yes | (M. J. Gauthier, 1977) |
| *P. distincta* ATCC700518 | No | (Bowman, 2007) |
| *P. elyakovii* ATCC700519 | No | (Sawabe et al., 2000) |
| *P. espejiana* ATCC29659 | No | (G. Gauthier, Gauthier, & Christen, 1995) |
| *P. flavipulchra* JG1 | Yes | (Yu et al., 2012) |
| *P. gelatinilytica* NH153 | No | (Yan et al., 2016) |
| *P. haloplanktis* ATCC14393 | No | (Holmström, Egan, Franks, McCloy, & Kjelleberg, 2002) |
| *P. issachenkonii* KCT12958 | No | (Ivanova, Sawabe, Alexeeva, et al., 2002) |
| *P. lipolytica* CGMCC1-8499 | No | (Xu et al., 2010) |
| *P. luteoviolacea* DSM6061 | Yes | (G. Gauthier et al., 1995) |
| *P. luteoviolacea* HI1 | Yes | (Asahina & Hadfield, 2015) |
| *P. marina* mano4 | No | (Nam et al., 2007) |
| *P. mariniglutinosa* KCTC22327 | No | (Romanenko, Zhukova, Lysenko, Mikhailov, & Stackebrandt, 2003) |
| *P. nigrifaciens* KMM661 | No | (G. Gauthier et al., 1995) |
| *P. phenolica* KCTC12086 | Yes | (Isnansetyo & Kamei, 2003) |
| *P. piscicida* ATCC15057 | Yes | (G. Gauthier et al., 1995) |
| *P. piscicida* DE2-B | Yes | (G. Gauthier et al., 1995) |
| *P. piscicida* JCM20779 | Yes | (Xie et al., 2012) |
| *P. piscicida* S2040 | Yes | (Machado, Sonnenschein, Melchiorsen, & Gram, 2015) |
| *P. piscicida* S2724 | Yes | (Machado et al., 2015) |
| *P. prydzensis* DSM14232 | No | (Bowman, 1998) |
| *P. rubra* ATCC29570 | Yes | (Neu, Månsson, Gram, & Prol-García, 2014) |
| *P. shioyasakiensis* JCM18891 | No | (Matsuyama et al., 2014) |
| *Pseudoalteromonas* sp. JC28 | Yes | This study |
| *P. tetraodonis* CSB01KR | No | (G. Gauthier et al., 1995) |
| *P. translucida* KMM520 | No | (Ivanova, Sawabe, Lysenko, et al., 2002) |
| *P. tunicata* D2 | Yes | (Holmstrom, James, Neilan, & White, 1998) |
| *P. undina* NCIMB2128 | No | (G. Gauthier et al., 1995) |
| *P. ulvae* TC14 | Yes | (Egan, Holmström, & Kjelleberg, 2001) |
| *V. harveyi* ATCC35084 | No | (Grimes et al., 1984) |

## References

Asahina, A., & Hadfield, M. (2015). Draft Genome Sequence of *Pseudoalteromonas luteoviolacea* HI1, Determined Using Roche 454 and PacBio Single-Molecule Real-Time Hybrid Sequencing. *Genome Announcements*, *3*(1), 4–5. https://doi.org/10.1128/genomeA.01590-14.Copyright

Bernbom, N., Ng, Y. Y., Kjelleberg, S., Harder, T., & Gram, L. (2011). Marine bacteria from Danish coastal waters show antifouling activity against the marine fouling bacterium *Pseudoalteromonas* sp. strain S91 and zoospores of the green alga *Ulva australis* independent of bacteriocidal activity. *Applied and Environmental Microbiology*, *77*(24), 8557–8567. https://doi.org/10.1128/AEM.06038-11

Bowman, J. P. (1998). Pseudoalteromonas prydzensis sp. nov., a psychotrophic, halotolerant bacterium from Antarctic sea ice. *International Journal of Systematic Bacteriology*, *48*, 1037–1041.

Bowman, J. P. (2007). Bioactive compound synthetic capacity and ecological significance of marine bacterial genus *Pseudoalteromonas*. *Marine Drugs*, *5*(4), 220–241. Retrieved from http://www.pubmedcentral.nih.gov/articlerender.fcgi?artid=2365693&tool=pmcentrez&rendertype=abstract

Egan, S., Holmström, C., & Kjelleberg, S. (2001). *Pseudoalteromonas ulvae* sp. nov., a bacterium with antifouling activities isolated from the surface of a marine alga. *International Journal of Systematic and Evolutionary Microbiology*, *51*(4), 1499–1504. https://doi.org/10.1099/00207713-51-4-1499

Gauthier, G., Gauthier, M., & Christen, R. (1995). Phylogenetic analysis of the genera *Alteromonas*, *Shewanella*, and *Moritella* using genes coding for small-subunit rRNA sequences and division of the genus *Alteromonas* into two genera, *Alteromonas* (emended) and *Pseudoalteromonas* gen. nov., and proposal of tw. *International Journal of Systematic Bacteriology*, *45*(4), 755–761. https://doi.org/10.1099/00207713-45-4-755

Gauthier, M. J. (1977). *Alteromonas citrea*, a New Gram-Negative, Yellow-Pigmented Species from Seawater. *International Journal of Systematic Bacteriology*, *27*(4), 349–354. https://doi.org/10.1099/00207713-27-4-349

Grimes, D. J., Stemmler, J., Hada, H., May, E. B., Maneval, D., Hetrick, F. M., … Colwell, R. R. (1984). *Vibrio* species associated with mortality of sharks held in captivity. *Microbial Ecology*, *10*(3), 271–282. https://doi.org/10.1007/BF02010940

Holmström, C., Egan, S., Franks, A., McCloy, S., & Kjelleberg, S. (2002). Antifouling activities expressed by marine surface associated *Pseudoalteromonas* species. *FEMS Microbiology Ecology*, *41*(1), 47–58. https://doi.org/10.1016/S0168-6496(02)00239-8

Holmstrom, C., James, S., Neilan, B. A., & White, D. C. (1998). *Pseudoalteromonas tunicata* sp. nov., a bacterium that produces antifouling agents. *International Journal of Systematic Bacteriology*, *48*, 1205–1212.

Isnansetyo, A., & Kamei, Y. (2003). *Pseudoalteromonas phenolica* sp. nov., a novel marine bacterium that produces phenolic anti-methicillin-resistant *Staphylococcus aureus* substances. *International Journal of Systematic and Evolutionary Microbiology*, *53*(2), 583–588. https://doi.org/10.1099/ijs.0.02431-0

Ivanova, E. P., Sawabe, T., Alexeeva, Y. V., Lysenko, A. M., Gorshkova, N. M., Hayashi, K., … Mikhailov, V. V. (2002). *Pseudoalteromonas issachenkonii* sp. nov., a bacterium that degrades the thallus of the brown alga *Fucus evanescens*. *International Journal of Systematic and Evolutionary Microbiology*, *52*(1), 229–234.

https://doi.org/10.1099/00207713-52-1-229
Ivanova, E. P., Sawabe, T., Lysenko, A. M., Gorshkova, N. M., Hayashi, K., Zhukova, N. V, … Atcc, T. (2002). *Pseudoalteromonas translucida* sp. nov. and *Pseudoalteromonas paragorgicola* sp. nov., and emended description of the genus. *International Journal of Systematic and Evolutionary Microbiology*, *52*, 1759–1766.
Machado, H., Sonnenschein, E. C., Melchiorsen, J., & Gram, L. (2015). Genome mining reveals unlocked bioactive potential of marine Gram-negative bacteria. *BMC Genomics*, *16*(1), 1–12. https://doi.org/10.1186/s12864-015-1365-z
Matsuyama, H., Minami, H., Kasahara, H., Kato, Y., Murayama, M., & Yumoto, I. (2013). *Pseudoalteromonas arabiensis* sp. nov., a marine polysaccharide-producing bacterium. *International Journal of Systematic and Evolutionary Microbiology*, *63*(PART 5), 1805–1809. https://doi.org/10.1099/ijs.0.043604-0
Matsuyama, H., Minami, H., Kasahara, H., Kato, Y., Murayama, M., & Yumoto, I. (2014). *Pseudoalteromonas shioyasakiensis* sp. nov., a marine polysaccharide-producing bacterium. *International Journal of Systematic and Evolutionary Microbiology*, *63*(PART 5), 1805–1809. https://doi.org/10.1099/ijs.0.043604-0
Nam, Y. Do, Chang, H. W., Park, J. R., Kwon, H. Y., Quan, Z. X., Park, Y. H., … Bae, J. W. (2007). *Pseudoalteromonas marina* sp. nov., a marine bacterium isolated from tidal flats of the Yellow Sea, and reclassification of *Pseudoalteromonas sagamiensis* as *Algicola sagamiensis* comb. nov. *International Journal of Systematic and Evolutionary Microbiology*, *57*(1), 12–18. https://doi.org/10.1099/ijs.0.64523-0
Neu, A. K., Månsson, M., Gram, L., & Prol-García, M. J. (2014). Toxicity of bioactive and probiotic marine bacteria and their secondary metabolites in *Artemia* sp. and *Caenorhabditis elegans* as eukaryotic model organisms. *Applied and Environmental Microbiology*, *80*(1), 146–153. https://doi.org/10.1128/AEM.02717-13
Park, Y. D., Bak, K. S., Yi, H., Bae, K. S., & Chun, J. (2005). *Pseudoalteromonas byusanensis* sp. nov., isolated from tidal flat sediment in Korea. *International Journal of Systematic and Evolutionary Microbiology*, *55*(6), 2519–2523. https://doi.org/10.1099/ijs.0.63750-0
Romanenko, L. A., Zhukova, N. V., Lysenko, A. M., Mikhailov, V. V., & Stackebrandt, E. (2003). Assignment of "*Alteromonas marinoglutinosa*" NCIMB 1770 to *Pseudoalteromonas mariniglutinosa* sp. nov., nom. rev., comb. nov. *International Journal of Systematic and Evolutionary Microbiology*, *53*(4), 1105–1109. https://doi.org/10.1099/ijs.0.02564-0
Romanenko, L. A., Zhukova, N. V., Rohde, M., Lysenko, A. M., Mikhailov, V. V., & Stackebrandt, E. (2003). *Pseudoalteromonas agarivorans* sp. nov., a novel marine agarolytic bacterium. *International Journal of Systematic and Evolutionary Microbiology*, *53*(1), 125–131. https://doi.org/10.1099/ijs.0.02234-0
Sawabe, T., Tanaka, R., Iqbal, M. M., Tajima, K., Ezura, Y., Ivanova, E. P., & Christen, R. (2000). Assignment of *Alteromonas elyakovii* KMM 162T and five strains isolated from spot-wounded fronds of *Laminaria japonica* to *Pseudoalteromonas elyakovii* comb. nov. and the extended description of the species. *International Journal of Systematic and Evolutionary Microbiology*, *50*(1), 265–271. https://doi.org/10.1099/00207713-50-1-265
Xie, B. Bin, Shu, Y. L., Qin, Q. L., Rong, J. C., Zhang, X. Y., Chen, X. L., … Zhang, Y. Z. (2012). Genome sequences of type strains of seven species of the marine bacterium *Pseudoalteromonas*. *Journal of Bacteriology*, *194*(10), 2746–2747. https://doi.org/10.1128/JB.00265-12

Xu, X.-W., Wu, Y.-H., Wang, C.-S., Gao, X.-H., Wang, X.-G., & Wu, M. (2010). *Pseudoalteromonas lipolytica* sp. nov., isolated from the Yangtze River estuary. *International Journal of Systematic and Evolutionary Microbiology*, *60*(Pt 9), 2176–2181. https://doi.org/10.1099/ijs.0.017673-0

Yan, J., Wu, Y.-H., Meng, F.-X., Wang, C.-S., Xiong, S.-L., Zhang, X.-Y., … Zhang, D.-M. (2016). *Pseudoalteromonas gelatinilytica* sp. nov., isolated from surface seawater. *International Journal of Systematic and Evolutionary Microbiology*, *66*(9), 3538–3545. https://doi.org/10.1099/ijsem.0.001224

Yu, M., Wang, J., Tang, K., Shi, X., Wang, S., Zhu, W. M., & Zhang, X. H. (2012). Purification and characterization of antibacterial compounds of *Pseudoalteromonas flavipulchra* JG1. *Microbiology*, *158*(3), 835–842. https://doi.org/10.1099/mic.0.055970-0
