# Supplementary material for: Hawaiian Bobtail Squid Symbionts Inhibit Marine Bacteria via Production of Specialized Metabolites, Including New Bromoalterochromides BAC-D/D′
Source: mSphere. 2020 Jul 1;5(4):e00166-20. doi: 10.1128/mSphere.00166-20 (PMC7333567; doi:10.1128/mSphere.00166-20)
Supplement: TABLE S3 [file mSphere.00166-20-st003.pdf]

Table S3a. Zone of inhibition areas of ANG/JC isolates tested against seven marine bacteria *in vitro*. Areas (cm^2) are an average of three trials ± the standard error of the mean

	ANG/JC Isolate	1[a]	2	3	4	5	6	7
Alphaproteobacteria	*Leisingera* sp. ANG1	0.0 ± 0	0.0 ± 0	0.0 ± 0	0.0 ± 0	0.0 ± 0	0.0 ± 0	0.0 ± 0
	Leisingera sp. ANG-M6	0.0 ± 0	0.0 ± 0	0.0 ± 0	0.0 ± 0	0.0 ± 0	0.0 ± 0	0.0 ± 0
	Leisingera sp. ANG-DT	0.0 ± 0	0.0 ± 0	0.0 ± 0	0.0 ± 0	0.0 ± 0	0.0 ± 0	0.0 ± 0
	Leisingera sp. ANG-S	0.0 ± 0	0.0 ± 0	0.0 ± 0	0.0 ± 0	0.0 ± 0	0.0 ± 0	0.0 ± 0
	Leisingera sp. ANG-S3	0.0 ± 0	0.0 ± 0	0.0 ± 0	0.0 ± 0	0.0 ± 0	0.0 ± 0	0.0 ± 0
	Leisingera sp. ANG52	0.0 ± 0	0.0 ± 0	0.0 ± 0	0.0 ± 0	0.0 ± 0	0.0 ± 0	0.0 ± 0
	Leisingera sp. ANG59	0.16 ± 0.01	0.25 ± 0.01	0.0 ± 0	0.0 ± 0	0.0 ± 0	0.0 ± 0	0.0 ± 0
	Leisingera sp. JC11	0.0 ± 0	0.0 ± 0	0.0 ± 0	0.0 ± 0	0.0 ± 0	0.0 ± 0	0.0 ± 0
	Rhodobacteraceae sp. ANG7	0.0 ± 0	0.0 ± 0	0.0 ± 0	0.0 ± 0	0.0 ± 0	0.0 ± 0	0.0 ± 0
	Rhodobacteraceae sp. ANG13	0.0 ± 0	0.0 ± 0	0.0 ± 0	0.0 ± 0	0.0 ± 0	0.0 ± 0	0.0 ± 0
	Labrenzia sp. ANG18	0.0 ± 0	0.0 ± 0	0.0 ± 0	0.0 ± 0	0.0 ± 0	0.0 ± 0	0.0 ± 0
	Ruegeria sp. ANG6	0.0 ± 0	0.0 ± 0	0.0 ± 0	0.0 ± 0	0.0 ± 0	0.0 ± 0	0.0 ± 0
	Ruegeria sp. ANG10	0.0 ± 0	0.0 ± 0	0.0 ± 0	0.0 ± 0	0.0 ± 0	0.0 ± 0	0.0 ± 0
	Ruegeria sp. ANG-S4	0.0 ± 0	0.0 ± 0	0.0 ± 0	0.0 ± 0	0.0 ± 0	0.0 ± 0	0.0 ± 0
Flavo.	*Muricauda* sp. ANG21	0.0 ± 0	0.0 ± 0	0.0 ± 0	0.0 ± 0	0.0 ± 0	0.0 ± 0	0.0 ± 0
Gamma.	*Shewanella* sp. ANG44	0.0 ± 0	0.0 ± 0	0.0 ± 0	0.0 ± 0	0.0 ± 0	0.0 ± 0	0.0 ± 0
	Alteromonas sp. JC21	0.0 ± 0	0.0 ± 0	0.0 ± 0	0.0 ± 0	0.0 ± 0	0.0 ± 0	0.0 ± 0
	Pseudoalteromonas sp. JC28	2.56 ± 0.61	3.21 ± 0.10	3.27 ± 0.43	3.86 ± 0.08	1.1 ± 0.07	2.28 ± 0.35	2.54 ± 0.19
	Vibrio sp. JC34	0.0 ± 0	0.0 ± 0	0.0 ± 0	0.0 ± 0	0.0 ± 0	0.0 ± 0	0.0 ± 0

Abbreviations: *Flavo.* = *Flavobacteriia*; *Gamma.* = *Gammaproteobacteria*

[a] Numbers refer to the following strains: 1, *Vibrio fischeri* ES114; 2, *Vibrio anguillarum* 775; 3, *Vibrio parahaemolyticus* KNH1; 4, *Vibrio harveyi* B392; 5, *Photobacterium leiognathi* KNH6; 6, *Bacillus megaterium* CNJ778; 7, *Exiguobacterium aestuarii* CNJ771

Table S3b. Zone of inhibition areas of *Pseudoalteromonas* sp. JC28 assayed against the target bacteria

Target strain	10^4 lawn density average area (cm^2)	10^5 lawn density average area (cm^2)	10^6 lawn density average area (cm^2)
P. leiognathi KNH6	1.63 ± 0.05	1.63 ± 0.05	0.24 ± 0.01
V. harveyi B392	3.85 ± 0.09	2.82 ± 0.12	1.10 ± 0.05
V. fischeri ES114	4.50 ± 0.15	2.91 ± 0.08	2.02 ± 0.25
V. parahaemolyticus KNH1	2.80 ± 0.07	2.14 ± 0.20	0.59 ± 0.04
V. anguillarum 775	11.1 ± 0.29	8.59 ± 0.33	4.72 ± 0.14
E. aesteuri CNJ 771	5.34 ± 0.13	4.65 ± 0.20	4.38 ± 0.08
B. megaterium CNJ 778	3.42 ± 0.27	2.55 ± 0.29	1.12 ± 0.05

Areas are an average of 9 trials ± the standard error of the mean.
