# Supplementary material for: Hawaiian Bobtail Squid Symbionts Inhibit Marine Bacteria via Production of Specialized Metabolites, Including New Bromoalterochromides BAC-D/D′
Source: mSphere. 2020 Jul 1;5(4):e00166-20. doi: 10.1128/mSphere.00166-20 (PMC7333567; doi:10.1128/mSphere.00166-20)
Supplement: TABLE S4 [file mSphere.00166-20-st004.pdf]

**Table S4.** Indigoidine biosynthetic genes detected in the *Leisingera* sp. ANG59 genome

| Gene | Function | % amino acid similarity to *igi* cluster in *Leisingera* sp. JC1 | E-value | % query cover | *Leisingera* sp. ANG59 locus ID |
|---|---|---|---|---|---|
| *igiR* | Transcriptional regulator, TetR family | 80.90 | 7E-94 | 100 | GKC28_24980 |
| *igiB* | Hydroxyisobutyrate dehydrogenase | 79.46 | 4E-148 | 100 | GKC28_24985 |
| *igiC* | Arylmalonate decarboxylase | 85.54 | 1E-142 | 100 | GKC28_24990 |
| *igiD* | Peptide synthetase | 83.91 | 0.0 | 99 | GKC28_24995 |
| *igiF* | 4-oxalocrotonate tautomerase | 75.32 | 1E-35 | 100 | GKC28_25000 |
| *igiE* | MFS transporter | 83.74 | 0.0 | 100 | GKC28_25005 |
