# Supplementary material for: Hawaiian Bobtail Squid Symbionts Inhibit Marine Bacteria via Production of Specialized Metabolites, Including New Bromoalterochromides BAC-D/D′
Source: mSphere. 2020 Jul 1;5(4):e00166-20. doi: 10.1128/mSphere.00166-20 (PMC7333567; doi:10.1128/mSphere.00166-20)
Supplement: TABLE S5 [file mSphere.00166-20-st005.pdf]

Table S5. Secondary metabolite biosynthetic gene clusters predicted by antiSMASH in the *Leisingera* sp. ANG59 and *Pseudoalteromonas* sp. JC28 genomes

Genome	Cluster type	Most similar known cluster	Scaffold	Total scaffold length (bp)	Cluster bp position	Total cluster length (bp)	% of scaffold containing cluster
***Leisingera* sp. ANG59**	Bacteriocin	NA	8.1	178,967	56,237 – 67,091	10,854	6.1
	Ectoine	Ectoine (100%)	72.1	13,164	3,162 – 12,740	9,578	72.8
	Siderophore	NA	43.1	31,755	15,090 – 26,979	11,889	37.4
	Other	NA	69.1	14,539	1 – 14,071	14,070	96.8
	Other	NA	13.1	125,897	94,639 – 121,837	27,198	21.6
	Siderophore	NA	79.1	9,946	1 – 8,467	8,466	85.1
	Homoserine lactone	NA	5.1	209,568	33,137 – 53,778	20,641	9.8
***Pseudoalteromonas* sp. JC28**	Ladderane-NRPS	Alterochromide (100%)	1.1	1,226,393	66,592 – 137,002	70,410	5.7
	Bacteriocin	NA	1.1	1,226,393	275,617 – 286,481	10,864	0.89
	T1PKS-NRPS	NA	1.1	1,226,393	633,994 – 683,565	49,571	4
	Thiopeptide	NA	1.1	1,226,393	789,245 – 826,848	37,603	3.1
	Bacteriocin	NA	3.1	509,001	116,400 – 127,955	11,555	2.3
	Bacteriocin	NA	3.1	509,001	209,010 – 220,206	11,196	2.2
	NRPS	NA	3.1	509,001	401,470 – 461,115	59,645	11.7
	NRPS	NA	6.1	374,113	316,144 – 372,250	56,106	15
	T1PKS-NRPS	Lotilibcin (20%)	7.1	531,598	1 – 86,834	86,834	16.3
	T1PKS-NRPS	NA	8.1	314,808	76,426 – 148,258	71,832	22.8
	NRPS	NA	11.1	183,335	159,352 – 183,335	23,983	13.1
	T1PKS-NRPS	NA	14.1	64,805	21,604 – 64,262	42,658	65.8
	NRPS	Cupriachelin (11%)	16.1	35,618	77 – 35,618	35,541	99.8
	NRPS	NA	21.1	11,311	1 – 11,311	11,311	100
	NRPS	NA	22.1	7,911	3,369 – 7,768	4,399	55.6
	NRPS	NA	24.1	4,435	1 – 4,435	4,435	100
	NRPS	NA	25.1	3,916	1-3,916	3,916	100
	NRPS	NA	28.1	1,164	1 – 1,164	1,164	100
