# Supplementary material for: Hawaiian Bobtail Squid Symbionts Inhibit Marine Bacteria via Production of Specialized Metabolites, Including New Bromoalterochromides BAC-D/D′
Source: mSphere. 2020 Jul 1;5(4):e00166-20. doi: 10.1128/mSphere.00166-20 (PMC7333567; doi:10.1128/mSphere.00166-20)
Supplement: TABLE S6 [file mSphere.00166-20-st006.pdf]

Table S6. Percent amino acid identity to bromoalterochromide gene cluster of BAC-containing *Pseudoalteromonas* genomes in MLSA tree (Fig. 2)

Strain	Gene	% amino acid identity	E-value	% query cover
P. elyakovii ATCC700519	*altA*	99.1	0	100.0
P. elyakovii ATCC700519	*altB*	100.0	5.13E-50	100.0
P. elyakovii ATCC700519	*altC*	97.2	0	100.0
P. elyakovii ATCC700519	*altD*	98.8	0	100.0
P. elyakovii ATCC700519	*altE*	100.0	5.11E-174	100.0
P. elyakovii ATCC700519	*altF*	100.0	0	100.0
P. elyakovii ATCC700519	*altG*	100.0	3.06E-86	100.0
P. elyakovii ATCC700519	*altH*	99.6	6.09E-149	91.8
P. elyakovii ATCC700519	*altI*	97.0	5.60E-153	100.0
P. elyakovii ATCC700519	*altJ*	98.4	2.78E-149	100.0
P. elyakovii ATCC700519	*altK*	98.7	0	97.8
P. elyakovii ATCC700519	*altL*	96.7	0	95.0
P. elyakovii ATCC700519	*altM*	98.2	0	79.2
P. elyakovii ATCC700519	*altN*	99.3	0	100.0
P. flavipulchra JG1	*altA*	99.6	0	100.0
P. flavipulchra JG1	*altB*	100.0	5.19E-50	100.0
P. flavipulchra JG1	*altC*	98.2	0	100.0
P. flavipulchra JG1	*altD*	97.8	0	100.0
P. flavipulchra JG1	*altE*	100.0	5.21E-174	100.0
P. flavipulchra JG1	*altF*	100.0	0	100.0
P. flavipulchra JG1	*altG*	100.0	3.10E-86	100.0
P. flavipulchra JG1	*altH*	99.1	1.39E-148	91.8
P. flavipulchra JG1	*altI*	96.2	1.30E-151	100.0
P. flavipulchra JG1	*altJ*	98.4	9.05E-151	100.0
P. flavipulchra JG1	*altK*	98.4	0	97.8
P. flavipulchra JG1	*altL*	97.1	0	88.6
P. flavipulchra JG1	*altM*	97.4	0	69.3
P. flavipulchra JG1	*altN*	99.3	0	100.0
Pseudoalteromonas sp. JC28	*altA*	99.6	0	100.0
Pseudoalteromonas sp. JC28	*altB*	100.0	5.22E-50	100.0
Pseudoalteromonas sp. JC28	*altC*	98.0	0	100.0
Pseudoalteromonas sp. JC28	*altD*	97.7	0	100.0
Pseudoalteromonas sp. JC28	*altE*	100.0	5.24E-174	100.0
Pseudoalteromonas sp. JC28	*altF*	100.0	0	100.0
Pseudoalteromonas sp. JC28	*altG*	100.0	3.12E-86	100.0
Pseudoalteromonas sp. JC28	*altH*	99.1	1.39E-148	91.8
Pseudoalteromonas sp. JC28	*altI*	95.7	9.58E-151	100.0
Pseudoalteromonas sp. JC28	*altJ*	98.0	2.54E-149	100.0
Pseudoalteromonas sp. JC28	*altK*	98.4	0	97.8

Pseudoalteromonas sp. JC28	*altL*	97.3	0	100.0
Pseudoalteromonas sp. JC28	*altM*	96.9	0	100.0
Pseudoalteromonas sp. JC28	*altN*	97.8	0	100.0
P. piscicida ATCC15057	*altA*	100.0	0	100.0
P. piscicida ATCC15057	*altB*	100.0	5.33E-50	100.0
P. piscicida ATCC15057	*altC*	100.0	0	100.0
P. piscicida ATCC15057	*altD*	100.0	0	100.0
P. piscicida ATCC15057	*altE*	100.0	4.99E-174	100.0
P. piscicida ATCC15057	*altF*	100.0	0	100.0
P. piscicida ATCC15057	*altG*	100.0	2.97E-86	100.0
P. piscicida ATCC15057	*altH*	100.0	1.27E-149	91.8
P. piscicida ATCC15057	*altI*	100.0	1.99E-157	100.0
P. piscicida ATCC15057	*altJ*	100.0	3.18E-152	100.0
P. piscicida ATCC15057	*altK*	100.0	0	97.8
P. piscicida ATCC15057	*altL*	99.1	0	98.6
P. piscicida ATCC15057	*altM*	87.5	0	99.0
P. piscicida ATCC15057	*altN*	100.0	0	100.0
P. piscicida DE2B	*altA*	99.1	0	100.0
P. piscicida DE2B	*altB*	100.0	9.52E-50	100.0
P. piscicida DE2B	*altC*	92.8	0	89.0
P. piscicida DE2B	*altD*	96.0	0	100.0
P. piscicida DE2B	*altE*	100.0	5.04E-174	100.0
P. piscicida DE2B	*altF*	100.0	0	100.0
P. piscicida DE2B	*altG*	100.0	2.99E-86	100.0
P. piscicida DE2B	*altH*	98.2	7.07E-147	91.8
P. piscicida DE2B	*altI*	97.0	2.08E-152	100.0
P. piscicida DE2B	*altJ*	95.6	1.19E-146	100.0
P. piscicida DE2B	*altK*	97.4	0	86.8
P. piscicida DE2B	*altL*	96.8	0	100.2
P. piscicida DE2B	*altM*	94.9	0	100.0
P. piscicida DE2B	*altN*	97.3	0	100.0
P. piscicida JCM20779	*altA*	100.0	0	100.0
P. piscicida JCM20779	*altB*	100.0	5.32E-50	100.0
P. piscicida JCM20779	*altC*	100.0	0	100.0
P. piscicida JCM20779	*altD*	100.0	0	100.0
P. piscicida JCM20779	*altE*	100.0	4.94E-174	100.0
P. piscicida JCM20779	*altF*	100.0	0	100.0
P. piscicida JCM20779	*altG*	100.0	2.96E-86	100.0
P. piscicida JCM20779	*altH*	100.0	1.25E-149	91.8
P. piscicida JCM20779	*altI*	100.0	1.97E-157	100.0
P. piscicida JCM20779	*altJ*	100.0	3.14E-152	100.0
P. piscicida JCM20779	*altK*	100.0	0	97.8
P. piscicida JCM20779	*altL*	100.0	0	91.9

P. piscicida JCM20779	*altM*	100.0	0	69.1
P. piscicida JCM20779	*altN*	100.0	0	100.0
P. piscicida S2040	*altA*	99.6	0	100.0
P. piscicida S2040	*altB*	99.0	3.31E-49	100.0
P. piscicida S2040	*altC*	97.6	0	100.0
P. piscicida S2040	*altD*	98.1	0	100.0
P. piscicida S2040	*altE*	100.0	4.68E-174	100.0
P. piscicida S2040	*altF*	100.0	0	100.0
P. piscicida S2040	*altG*	99.2	1.35E-85	100.0
P. piscicida S2040	*altH*	100.0	1.19E-149	91.8
P. piscicida S2040	*altI*	97.9	1.34E-153	100.0
P. piscicida S2040	*altJ*	98.4	1.63E-150	100.0
P. piscicida S2040	*altK*	99.1	0	97.8
P. piscicida S2040	*altL*	98.0	0	80.9
P. piscicida S2040	*altM*	97.5	0	67.6
P. piscicida S2040	*altN*	99.0	0	100.0
P. piscicida S2724	*altA*	99.6	0	100.0
P. piscicida S2724	*altB*	100.0	4.90E-50	100.0
P. piscicida S2724	*altC*	97.8	0	100.0
P. piscicida S2724	*altD*	98.2	0	100.0
P. piscicida S2724	*altE*	99.6	1.93E-173	100.0
P. piscicida S2724	*altF*	99.7	0	100.0
P. piscicida S2724	*altG*	100.0	2.92E-86	100.0
P. piscicida S2724	*altH*	99.1	2.09E-148	91.8
P. piscicida S2724	*altI*	96.2	4.38E-152	100.0
P. piscicida S2724	*altJ*	98.0	2.28E-150	100.0
P. piscicida S2724	*altK*	98.7	0	97.8
P. piscicida S2724	*altL*	96.9	0	100.2
P. piscicida S2724	*altM*	97.7	0	100.0
P. piscicida S2724	*altN*	99.0	0	100.0
