# Supplementary material for: Hawaiian Bobtail Squid Symbionts Inhibit Marine Bacteria via Production of Specialized Metabolites, Including New Bromoalterochromides BAC-D/D′
Source: mSphere. 2020 Jul 1;5(4):e00166-20. doi: 10.1128/mSphere.00166-20 (PMC7333567; doi:10.1128/mSphere.00166-20)
Supplement: TABLE S7 [file mSphere.00166-20-st007.pdf]

**Table S7**. Alterochromides detected by LC-MS in the EtOAc and 90% aqueous MeOH extracts of *Pseudooalteromonas* sp. JC28.

| $t_R$ (min) | Observed *m/z* | Remarks |
|---|---|---|
| 5.24 | 766.2 | Alterochromide A/A' |
| 7.71 | 844.2 | Bromoalterochromide A/A' |
| 8.86 | 858.2 | Bromoalterochromide D/D' |
| 9.83 | 870.2 | Bromoalterochromide B/B' |
| 10.27 | 924.1 | Dibromoalterochromide A/A' |
| 10.87 | 884.3 | Bromoalterochromide B/B' + $CH_2$ |
| 12.43 | 950.2 | Dibromoalterochromide B/B' |
